# Hydrothermal fabrication, characterization and RSM optimization of cobalt-doped zinc oxide nanoparticles for antibiotic photodegradation under visible light

**DOI:** 10.1038/s41598-024-52430-8

**Published:** 2024-01-23

**Authors:** Asmaa I. Meky, Mohamed A. Hassaan, Howida A. Fetouh, Amel M. Ismail, Ahmed El Nemr

**Affiliations:** 1 Department of Chemistry, Faculty of Science, Alexandria University, Alexandria, Egypt; 2 Environment Division, National Institute of Oceanography and Fisheries (NIOF), Kayet Bey, Elanfoushy, Alexandria, Egypt

**Keywords:** Environmental chemistry, Pollution remediation

## Abstract

Photodegradation is considered a significant method engaged for the elimination of organic pollutants from water. In this work, hydrothermal cobalt-doped zinc oxide nanoparticles (Hy-Co–ZnO NPs) loaded with 5, 10, and 15% cobalt were prepared in a hydrothermal way and were investigated as a photocatalyst for the Ciprofloxacin (CIPF) degradation under visible irradiation using LED-light. Characterization approaches such as FTIR, XRD, XPS, DRS UV–vis spectroscopy, SEM, TEM, BET, EDX and TGA were used for the investigation of the fabricated Hy-Co–ZnO NPs. The studies indicated that 10% Hy-Co–ZnO NPs was the most efficient catalyst for the CIPF photolysis compared to ZnO NPs and other Hy-Co–ZnO NPs with 5 and 15% cobalt content. Higher photocatalytic activity (> 98%) of 20 mg/L of CIPF solution was attained within 60 min. The reaction kinetics showed that the first-order model is suitable for displaying the rate of reaction and amount of CIPF elimination with *R*^2^ = 0.9883. Moreover, Central composite design (CCD) optimization of the 10% Hy-Co–ZnO NPs was also studied.

## Introduction

Ecosystems and human health have been seriously threatened by the widespread use of antibiotics^1,2^. The typical second-generation fluoroquinolone (FQ) antibiotic ciprofloxacin (CIPF) is widely used in both human and veterinary medicine because of its extensive antimicrobial activity and advantageous oral absorption characteristics^3^. Regrettably, the CIPF that enters a person or animal's body can be partially broken down by metabolism and nevertheless remain present in substantial concentrations in the natural environment in a pharmacological form^4^. Several environmental matrices have been identified to contain CIPF recently^5,6^. Additionally, most wastewater treatment facilities would not successfully remove it. Therefore, it is still essential to look for an effective method of removing CIPF from wastewater^1^. Indirect photolysis is the only abiotic breakdown process in surface water that is guaranteed to occur when pharmaceutical compounds are present^7,8^. So, advanced oxidation techniques (AOP) were used to degrade these emerging pollutants (CIPF)^9^. Heterogeneous photocatalysis is a successful, environmentally safe, and economically efficient AOP for the destruction of hazardous chemicals^7^.

One of the best techniques for treating water is photocatalysis by semiconductor nanostructures in the existence of light^10^. By lowering their band-gap energy, some highly effective photocatalysts including ZnO, TiO_2_, CeO_2_ and SnO_2_ can be used in this technique to shift their photocatalytic effectiveness towards visible light^10–13^. One of the photocatalysts that has been investigated the most is ZnO NPs in particular^14,15^. UV light can cause valence electrons to move onto the conduction band since the band gap energy of bulk ZnO NPs is 3.3 eV. Superoxide (·O^2−^) and hydroxyl (·OH), which are extremely reactive free radicals, are produced when these photo-excited electrons and holes migrate to the particle surfaces where water and oxygen molecules are present. These free radicals then participate in secondary processes including the breakdown of organic compounds. There has been a lot of effort done to enhance the photocatalytic capabilities of ZnO NPs^16^, including size reduction, facet engineering, photosensitization with dyes or quantum dots, surface ornamentation with charge separators, and impurity activation^14^.

These semiconductors' band gap energy can be lowered by using a select few dopants, including B, C, F, N, and S^10,16^. Another strategy to boost photocatalysis is to dope elements like Fe, Cu, Au, Pd, Pt, Ag, and Co with these semiconductor nanoparticles to lessen their ability to form the Schottky barrier^17^. Since cobalt has great solubility and a rich electron status, it is believed to be an appropriate metal for doping the matrix of ZnO^18,19^. Co^2+^ ions can easily replace Zn^2+^ ions in the lattice of ZnO because cobalt's ionic radius is close to that of zinc^20^. In the ZnO matrix, the imperfect d orbitals of CO ions act as electron traps, increasing the photogenerated charge separation. Cobalt doping is incorporated into the bandgap, creating new energy levels that may be activated by visible light^21^. This is related to the interaction between localized d electrons of transition metal ions and ZnO band edge electrons known as the sp-d spin exchange interaction^22^. Researchers have successfully established methods for synthesizing semiconductor nanomaterial in a variety of shapes, including nano-spheres, nanowires, and nanobelts^23–26^. Numerous techniques, such as the sol–gel method, vapor phase growth, solvothermal method, thermal decomposition method, combustion method, and hydrothermal method are used to create ZnO nanostructured thin films^27,28^. Due to its low cost, low temperature, low cost, and environmental friendliness, hydrothermal is considered to be the most effective method for creating ZnO nanorods^23^.

In the previous works, the rhodamine B dye degradation in water was achieved by using 10% cobalt doping ZnO NPs powder, which is an effective catalyst visible light that achieved removal by 93% within 120 min^18^. Direct growth of ZnO nanorods doped by 0, 3, 5, and 7 mol% Co on a glass substrate was achieved by using a straightforward spray pyrolysis process followed by the hydrothermal method. This study found that, in the presence of ZnO nanorods photocatalysts with 0, 3, 5, and 7 mol% Co, the rates of methyl blue degradation were, respectively, 67, 72, 78, and 80%^29^. Zhang et al.^30^ studied the impact of cobalt doping on ZnO samples. The 95 °C-prepared 2% Co^2+^ doped ZnO nanoarray showed high photocatalytic activity and could destroy 96% of methylene blue solution in 120 min while exposed to visible light. According to Pan et al.^31^, the Zn_0.96_Co_0.04_O sample displayed an improved efficiency of methylene blue photocatalytic decomposition compared with ZnO NPs.

The response surface methodology (RSM), a group of mathematical and statistical techniques for developing experimental models, includes the estimate of function and the experimental design as two of its major components. In the experimental trials, RSM was effectively used. RSM is used to reduce the cost of analytical procedures and associated numerical noise^32^. In the authors' knowledge, this is the first work that uses RSM-CCD for optimizing photocatalytic degradation of CIPF using Co-doped ZnO. In the present work, Co-doped ZnO NPs are formed by hydrothermal procedure, and the structural, morphological, and optical properties of the Hy-Co–ZnO NPs were studied. Additionally, the photocatalytic activity of the photocatalyst in its produced form was examined for the CIPF degradation process.

## Materials and method

### Materials

Cobalt acetate tetrahydrate, NaOH, Zinc acetate dihydrate, HCl, isopropanol, Na-EDTA and benzoquinone were purchased from Sigma Aldrich. CIPF was purchased as CIPF 200 mg/100 mL I.V. infusion solution from Amirya Pharmaceuticals, Egypt.

### Equipment and characterization techniques

The following instruments were applied to identify the samples of ZnO NPs and Hy-Co–ZnO NPs photocatalysts. ZnO NPs and Hy-Co–ZnO NPs crystallinity, and average crystal size were confirmed by Bruker Meas Srv (D2-diffractometer that controls at 30 kV, 10 mA using Cu tube *λ* = 1.5418 Å and 2*θ* with a temperature range of 5 to 80°) were used. Fourier transform infrared (FTIR) spectroscopy model VERTEX70 linked to platinum ATR V-100 model, Bruker, Germany, in the 400–4000 cm^−1^ wavenumber range. SEM (SEM-JEOL, IT 200 Japan) equipped with Energy dispersive X-ray spectroscopy (EDX) for elemental analysis was used to determine the materials' morphology and surface characteristics. TEM (JTM 1400 plus Japan) was used to determine the size and shape of the nanostructures. UV–Visible, GBC Cintra 3030 at the range 190–900 nm spectrophotometer was used to measure the optical absorbance of these samples. Using the BELSORP—Mini II from BEL Japan, Inc., the average pore diameter and specific surface area were measured using the BET (Brunauer–Emmett–Teller) model. The SDT650-Simultaneous Thermal analyzer equipment was applied to make thermal analyses for prepared samples utilizing a 10 °C per/min as a ramping temperature. XPS analyses were made using K-ALPHA (Thermo Fisher Scientific, USA) with monochromatic X-ray Al K-alpha radiation − 10 to 1350 eV spot size 400 µm under 10^−9^ mbar pressure with pass energy 200 eV full spectrum at 50 eV narrow spectrum.

### Preparation of ZnO and Co-doped ZnO (Zn_1−x_Co_x_O)

Following the addition of Solution-A (Zn (CH_3_COO)_2_.2H_2_O) (0.5 M) to 100 mL of distilled water, which was continuously stirred at 60 °C for 30 min, 0.5 M NaOH was added dropwise until the pH reached 12 and a white tint appeared. The solution was vigorously stirred for 1 h at the same temperature. The solution combination was held for 30 min for ultrasonication treatment in an ultrasonic water bath before being transferred to a Teflon-lined autoclave. The hydrothermal syntheses were carried out in an electric oven at 150 °C for 12 h. The obtained white precipitate was then separated, filtered, rinsed with distilled water and EtOH several times, dried, and heated to 500 °C^13,15^. Hy-ZnO NPs' whitish powder was carefully gathered and stored till needed. The cobalt doping ZnO (Zn_1-*x*_Co_*x*_O) NPs were synthesized via the hydrothermal method in the same way. Where different ratios (5, 10, 15% of Co/ZnO) were synthesized by dissolving the desired amount of Co (CH_3_COO)_2_·4H_2_O (solution-B) in distilled water under stirring for 30 min. Then (solution-B) was added dropwise to the zinc acetate (solution-A) followed by the same procedures of Hy-ZnO NPs synthesis procedures^15^.

### Photocatalytic activity

To determine the best catalyst performance, a specific amount of 100 mg of Hy-ZnO NPs and 5, 10, and 15% of Hy-Co–ZnO NPs was added to a Pyrex glass beaker containing 100 mL of CIPF with concentrations of 30 ppm at neutral pH for 2 h under (150 W LED light lamp) as visible light from LED source and the removal efficiency was measured. The photoreactor used for CIPF degradation was made up of a 150 W visible-LED-light source (wavelength range from 405 to 800 nm). In a typical photocatalytic degradation experiment, the photocatalyst was added to CIPF solution in a 100 mL flask beaker, and the mixture was left in the dark for 30 min to create the adsorption–desorption equilibrium. The reaction medium containing the photocatalyst and CIPF solution was then exposed to visible light. By taking 2 mL of the aliquot CIP solution at regular time intervals. A UV–vis spectrophotometer (model Pg/T80 UV/ Vis) was used for CIPF concentration analysis at a wavelength of λ 270 nm after it had been centrifuged at 6,000 rpm for 30 min. The degradation efficiency was obtained from Eq. (1).

1 $$Degradation \;efficiency=\frac{{C}_{0}-{C}_{t}}{{C}_{0}}\times 100$$

In which *C*_0_ stands as the CIPF primary concentration in water, and *C*_t_ refers to the CIPF concentration in the reaction mixture at definite time intervals of irradiation. Photolysis parameters were optimized for pH, catalyst doses, antibiotic concentrations, temperature, and shaking speed to discover the best conditions for efficient photolysis.

### Radical scavenger

Three scavengers (10 mM Na-EDTA, 1 mM IPA, and 1 mM BQ, individually) were added to a 100 mL, 30 ppm CIPF solution to quench the photo-generated species (holes (h^+^), hydroxyl radicals (·OH), and superoxide radicals (·O_2_^−^), which are each responsible for catalytic degradation, respectively)^33^.

### Design and model of experiments based on central composite

Response surface methodology (RSM) is applied to analyze the impacts of reaction speed, pH, initial CIPF concentration, and dose of 10% Hy-Co–ZnO NPs on CIPF degradation. RSM is a tool that integrates statistical and mathematical methods that can be applied to plan experiments, examine parameter relationships, and enhance procedures. The most widely used approach for creating environmental processes in RSM is central composite design (CCD), which permits a thorough understanding of how various components interact with one another with fewer trial runs (Table 1)^34,35^. A central composite rotatable design with 30 runs was applied to the independent variables. The empirical-second-order polynomial regression model was defined using the findings of the experimental technique, and is shown in the following Eq. (2) ^32^:

2 $$Y={\beta }_{0}+{\sum }_{j=1}^{k}\beta jxj+{\sum }_{j=1}^{k}\beta jjxj2+\sum {\sum }_{i<j}^{k}\beta i\beta jxixj$$

where *Y* is the outcome; *xi* and *xj* are independent variables (*i* and *j* varied from 1 to *k*); *β*_0_ is the constant term; *βj* is the linear coefficient; *βij* is the interaction coefficient; *βjj* is the quadratic coefficient; and *k* is the number of independent variables (k = 4 in this research)^32^.

**Table 1:** Low and high-level values for independent variables.

Factor	Parameter	Units	Minimum	Maximum	Mean	Std. Dev. ±
A	Catalyst dosage	Mg	20	100	60.00	18.19
B	Antibiotic dosage	mg/L	10	50	30.00	9.10
C	Shaking speed	RPM	50	250	150.00	45.49
D	pH		3	11	7.00	1.82

## Result and discussion

### FTIR analysis

**Figure 1:**
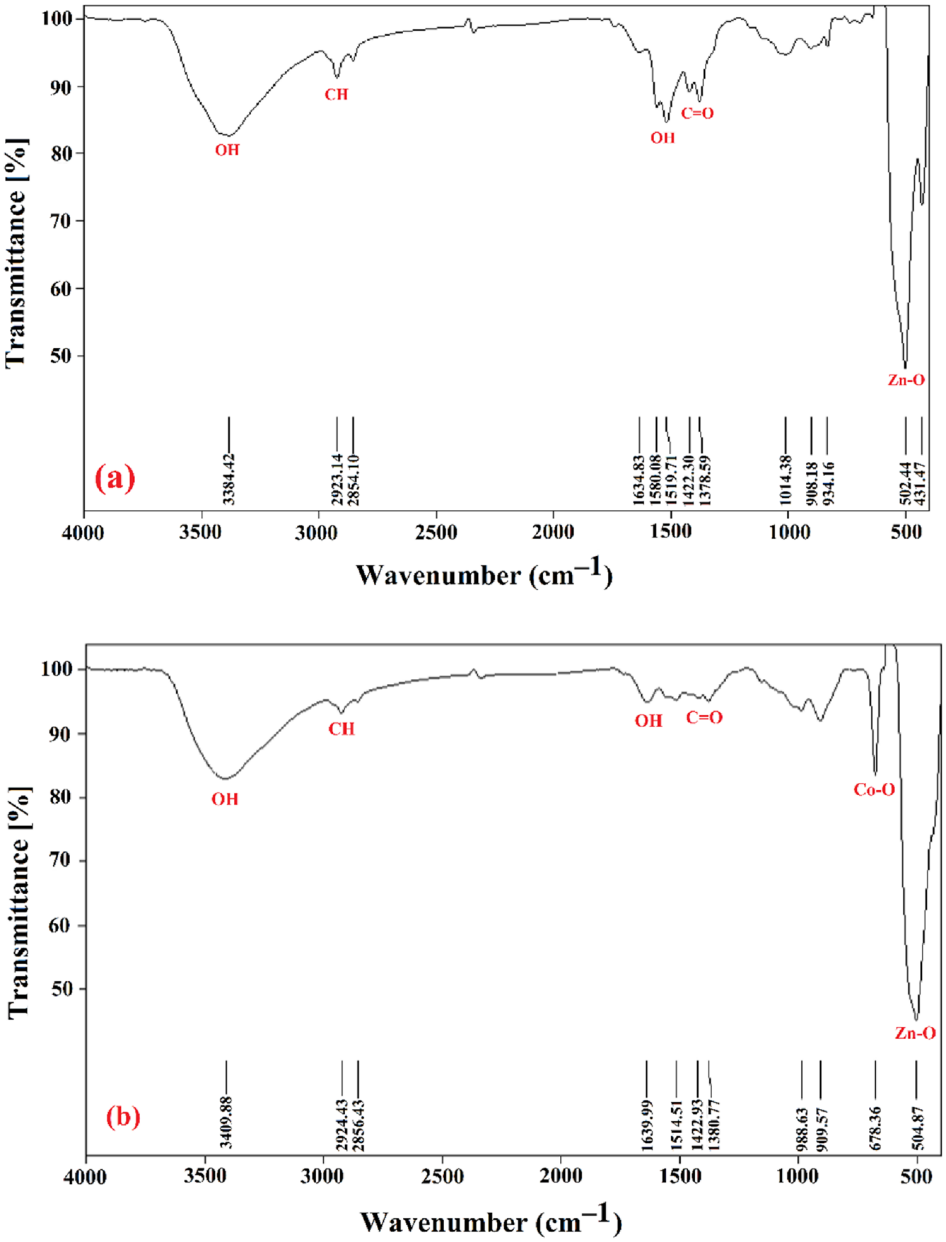
The FTIR studies of (**a**) Hy-ZnO NPs, and (**b**) 10% Hy-Co–ZnO NPs.

Figure 1 displays the FTIR spectra of Hy-ZnO NPs and Hy-Co–ZnO NPs powders (dry and annealed) recorded in the region 4000–400 cm^−1^. The Hy-Co–ZnO NPs samples’ FT-IR spectra made clear the sample's functional groups. The Co^2+^-doped samples displayed absorption peaks at about 678 cm^−1^ in comparison to pure Hy-ZnO NPs due to the IR absorption of Co–O^30,36, 37^, which further supports the integration of Co^2+^ into the ZnO lattice. Zn–O vibrations were attributed to the absorption band at 504 cm^−1^^38^. The stretching vibration of C=O was visible in the strong absorption bands between 1380 cm^−1^ and 1514 cm^−1^. The O–H stretching vibration mode was confirmed by the absorption bands at 1639 cm^−1^ and 3409 cm^−1^, which demonstrated the absorption of water from the surrounding environment^30^.

The vibration of acetate CH_2_ (C–H) groups can be seen in the absorption bands at 2856–2924 cm^−1^. ^39,40^. The weak band seen between 909 and 988 cm^−1^ is attributed to a change in metal–oxygen vibrational frequency caused by the accumulation of Co into the ZnO matrix^39^. Switching the band position of the ZnO bands towards higher values of wavenumber exposes that the Zn–O–Zn system is disturbed by the presence of in situ Co^41^. This switch is shown at the position of the Zn–O stretching peak with Co doping in Fig. 1.

From the IR spectra of the Hy-ZnO NPs sample, the band performed at 3384 cm^−1^ is allocated to the OH mode of the H_2_O molecule and the absorption peak observed at 869–880 cm^−1^ corresponds to the C–OH group. The absorption peak for Zn–O stretching is detected at 431 cm^−1^ for Hy-ZnO NPs^42^.

### BET analysis

In Figure S1, the pore size distribution plot and nitrogen adsorption/desorption isotherms of the Hy-ZnO and Hy-Co–ZnO NPs are displayed. Hy-ZnO NPs display the type IV curve according to the IUPAC classification, which is explained by the prevalence of mesopores^43^. The findings of the BET study show that the surface area, monolayer volume, total pore volume, and mean pore diameter of Hy-ZnO are, respectively, 8.3923 m^2^/g, 1.9282, 0.022861 m^3^/g, and 10.896 nm (Table 2, Table S1, and Fig. S1). 10% Hy-Co–ZnO had a specific surface area of 10.0053 m^2^/g, a monolayer volume of 2.3098 cm^3^/g, a total volume of 0.030428 cm^3^/g, and a mean pore diameter (*P*_m_) of 12.107 nm, respectively. Integrated pore volumes (*V*_p_) of Hy-ZnO and 10% Hy-Co–ZnO in the BJH adsorption analysis were 0.0222947 and 0.030277 cm^3^/g and mesopore-specific surface areas (8.5805 and 10.351 m^2^/g), respectively.

**Table 2:** Analysis of the surface area of Hy-ZnO NPs and Hy-Co–ZnO NPs.

	ZnO	10%Co–ZnO
BET		
*a*_s, BET_ (m^2^∕g)	8.3923	10.053
*V*_m_ (cm^3^ STP)/g)	1.9282	2.3098
Mean pore diameter *P*_m_ (nm)	10.896	12.107
The volume of total pore *V*_T_ (cm^3^/g)	0.022861	0.030428
BJH		
*V*_p_ (cm^3^/g)	0.022947	0.030277
*a*_p_ (m^2^/g)	8.5805	10.351

### Scanning electron microscope (SEM)

**Figure 2:**
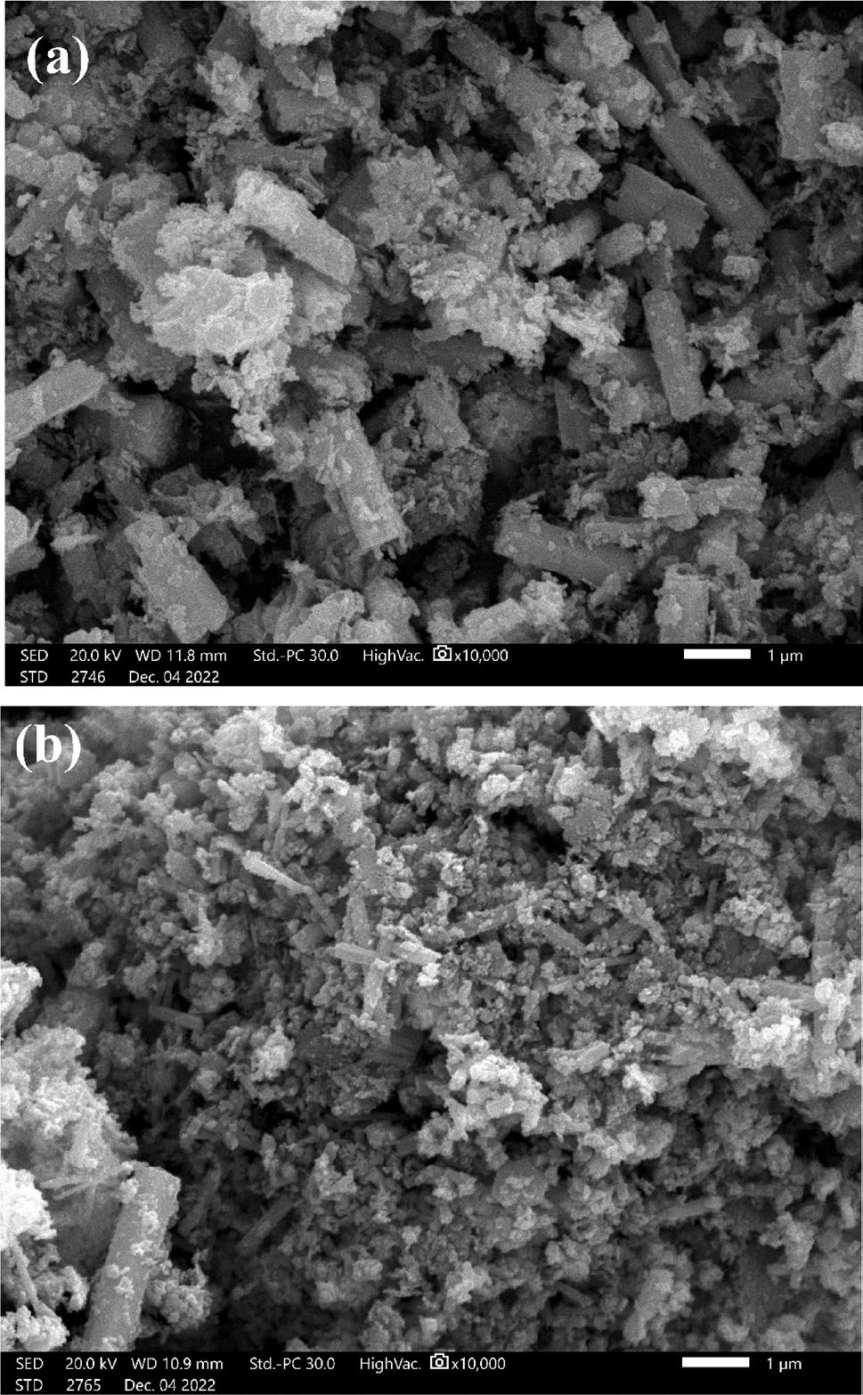
SEM pictures of (**a**) Hy-ZnO NPs, (**b**) 10% Hy-Co–ZnO NPs.

In the current work, SEM images of 10% Hy-Co-doped and pure Hy-ZnO nanorods are shown in Fig. 2a,b. Using a common magnification of 10,000, voids can be seen in the SEM picture as the morphology of the prepared samples was simultaneously captured. Images of pure and co-doped ZnO samples demonstrate a one-dimensional nanostructure, like a cuboid. The huge surface area and small particle size are essential to the increased photocatalytic activity^30^. Due to their huge surface area, one-dimensional nanostructures of cuboids are the best substances for improving photocatalytic activity. The Co ions insertion into ZnO lattice positions may have an impact on how the morphology of the cuboid evolves to become bounded grains. Due to the doping of Co, the tiny particles have aggregated and become linked to an irregular shape, Fig. 2b.

### EDX analysis

**Figure 3:**
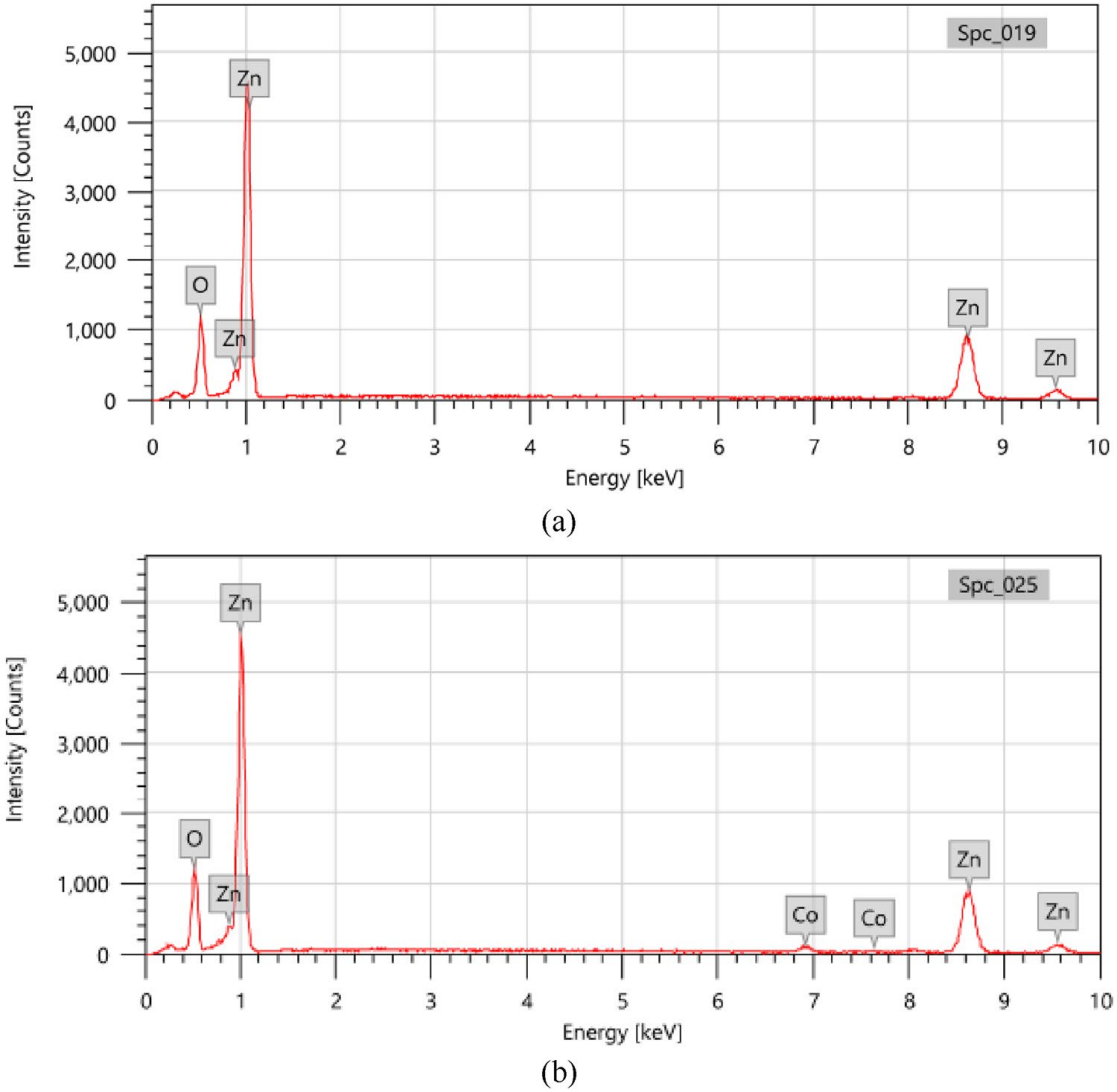
EDX bands of (**a**) Hy-ZnO NPs and (**b**) 10% Hy-Co–ZnO NPs.

The elemental makeup of 10% Hy-Co–ZnO NPs and Hy-ZnO NPs in the synthesized photocatalyst was investigated using an Energy Dispersive X-ray Diffractive (EDX) examination. Figure 3a displays how EDX confirmed the signals for zinc and oxygen coming from zinc oxide nanoparticles. In addition, the analysis discovered the peaks related to the optical absorbance of the synthesized nanoparticle. The nanoparticle's elemental analysis revealed 78.78 ± 87% zinc and 21.22 ± 25% oxygen, demonstrating that it was produced using the purest process possible (Table 3). If the percentage of Hy-Co–ZnO NPs is 10% according to Fig. 3b elemental analysis, the nanoparticle contains 2.76 ± 12 cobalt, 21.63 ± 24 oxygen, and 75.61 ± 85 zinc.

**Table 3:** Element analysis of ZnO and 10%Co–ZnO using EDX analysis.

Element	Hy-ZnO NPs		10% Hy-Co–ZnO NPs	
	Mass%	Atom%	Mass%	Atom%
Zn	78.78 ± 0.87	47.60 ± 0.53	75.61 ± 0.85	45.26 ± 0.51
O	21.22 ± 0.25	52.40 ± 0.61	21.63 ± 0.24	52.91 ± 0.60
Co	0	0	2.76 ± 0.12	1.83 ± 0.08
Total	100	100	100	100

### Transmission electron microscopic (TEM)

**Figure 4:**
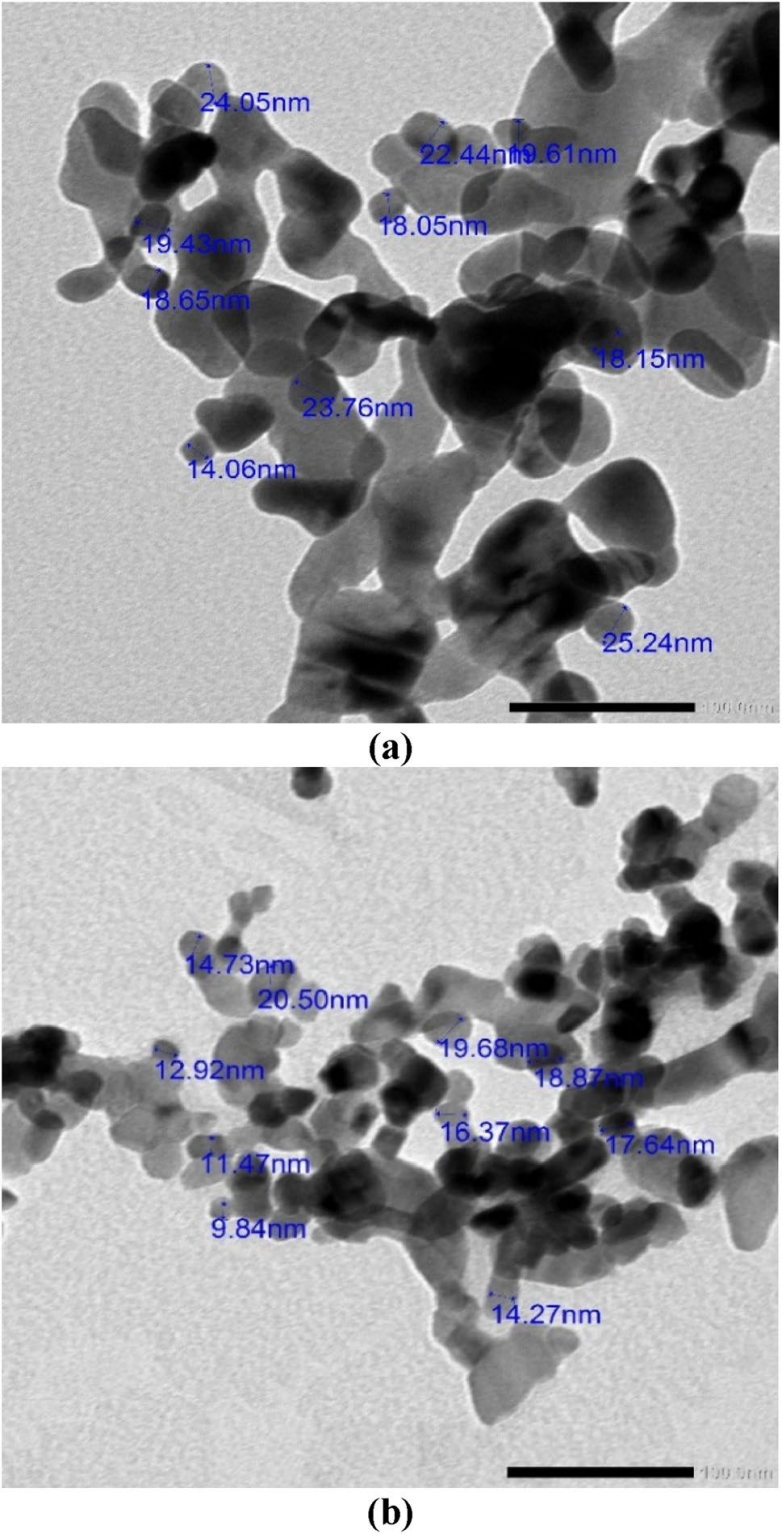
TEM pictures of (**a**) Hy-ZnO NPs, (**b**) 10% Hy-Co–ZnO NPs.

The measured values and the size of the nanostructure as established by transmission electron microscopy examination were consistent. Figure 4 shows TEM images of Hy-ZnO NPs and Hy-Co-doped ZnO NPs. The diagram makes it abundantly evident that the diameter of the created Co-doped sample is smaller than that of the Hy-ZnO NPs sample, but the length is essentially unaltered.

### X‑ray diffraction (XRD) study

**Figure 5:**
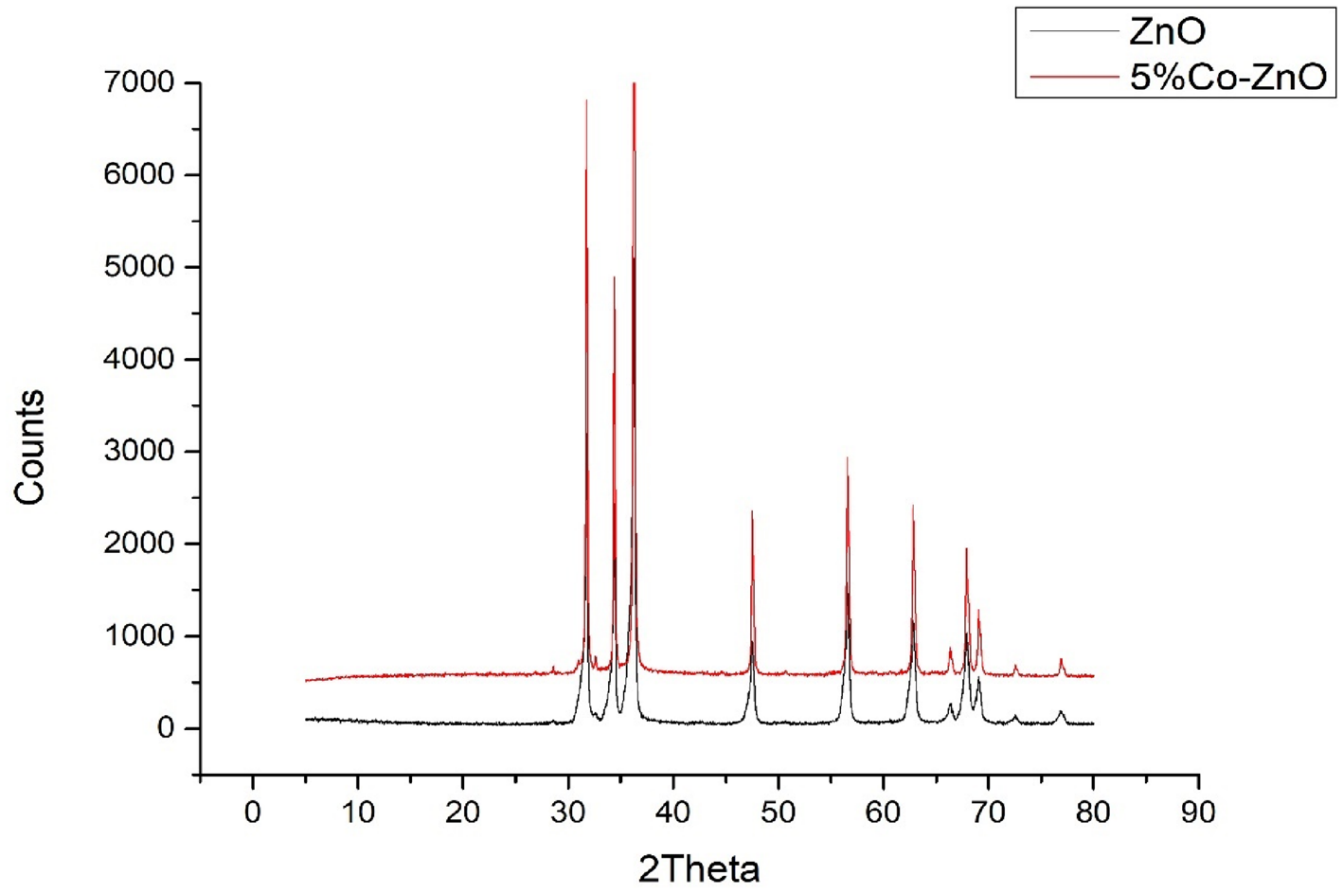
XRD analyses of (**a**) Hy-ZnO NPs, (**b**) 10% Hy-Co–ZnO NPs.

Figure 5 and Figure S2 show the powder X-ray diffractogram of the formed Hy-ZnO NPs and Hy-Co–ZnO NPs with various amounts of Co^2+^ ions, there are concentrations (in molar%) of Co^2+^ ions of 5, 10, and 15% (Fig. S2). All of the samples had a strong diffraction peak, which indicated that they were all extremely crystalline and only in the wurtzite structure^44,45^. Additionally, the fact that there is no sign of any other secondary phases, such as cobalt oxides, metallic cobalt, or binary zinc cobalt phases, shows that the samples are entirely single phases. Cobalt appears to function as a substitution dopant since samples of cobalt-doped ZnO NPs lack an impurity phase^44^. It should be noted that the three major XRD peaks match the crystal planes of hexagonal Zinc oxide (100), (101) and (002)^46^. Figure S2 shows that by increasing the doping concentration, the peak intensity rose. Additionally, a minor shift in the location of the ZnO (101) peak was seen when the amount of doped cobalt incorporated into the ZnO network increased^47^.

Figure 5 displays the enlarged XRD patterns of Hy-ZnO NPs and Hy-Co–ZnO NPs in the 10°–90° range. With increasing cobalt % in Hy-ZnO NPs, the position of the peaks is visibly shifting and enlarging toward a higher 2θ value. Since Co^2+^ has a smaller radius (0.65) than Zn^2+^ (0.74), moving the 2θ position of XRD patterns reveals a slight change in the lattice properties of ZnO with cobalt doping. The average crystal size of the synthesized catalyst was measured by the Debye–Scherrer Eq. (3) ^47^.

3 $$D=\frac{K\lambda }{\beta Cos\theta }$$

where *λ* stated for the X-ray wavelength (1.54178); *K* is stated for the shape factor (*K* = 0.94) and *Ө* stated for the Bragg angle, and *β* stated for the corrected line broadening is defined as full width at half maximum (FWHM).

The size of the crystallite grows from 46.07 to 67.30 nm as a result of the mismatch between the ionic radii of Zn^2+^ and Co^2+^ (Table S2). Since the lattice volume grows as a result of the existence of interstitial atoms at greater cobalt concentrations the average crystal size also increases^48^.

### UV–vis and DRS analysis

The green-colored Hy-Co–ZnO NPs exhibit significant visible absorption bands in the 550–700 nm region (near red–orange absorption coupled with blue–green appearance) and band gap displacement to lower energies (redshift). Using the following Eq. (4), the samples’ optical band gap energy (Eg) was calculated.

4 $$E=\frac{1240 }{\lambda }$$

With increasing cobalt concentrations to 10%, it is seen that the cobalt-doped ZnO NPs band gap energy gradually decreases.

Hy-ZnO NPs have a wavelength of 376 nm (3.29eV), whereas Co–ZnO exhibits Three absorption visible bands shown in the transmission spectra 565 nm (2.194eV), 615 nm (2.016 eV), and 663 nm (1.87 eV), which corresponds to the primary absorption peaks of the Co^2+^ tetrahedral molecular ions^49^.

**Figure 6:**
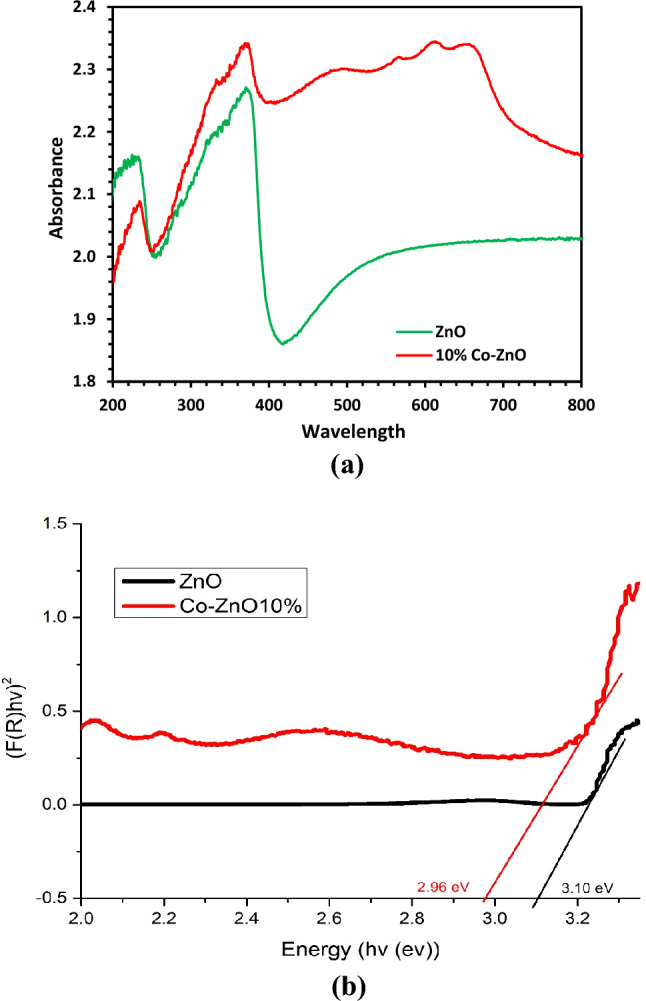
UV–Drs analyses of (**a**) Hy-ZnO NPs, and 10% Hy-Co–ZnO NPs and (**b**) obtained bandgap energy for Hy-ZnO NPs, and 10% Hy-Co–ZnO NPs through the K-M function.

Figure 6 indicates that the position of the absorption spectra shifts towards the red with increasing Co-doping concentration in ZnO, indicating that the band gap of ZnO materials reduces with increasing concentration of Co-doping in ZnO. The redshift of cobalt-doped ZnO NPs is frequently caused by the *sp-d* exchange interaction between the ZnO band electrons and localized *d* electrons of Co^2+^ ions replacing Zn^2+^ ions. As a result of these exchange interactions, the positive and negative energy bands are corrected, resulting in a band gap narrowing^44,50^.

The optical band gap Eg was calculated by applying the Kubelka–Munk (K-M) method to the reflectance (R) data^44^. The K-M method is based on the following equation:

5 $$F(R)=\frac{{(1-R)}^{2}}{2R}$$

The K-M function (F(R)) is proportional to the absorption coefficient (∝). Therefore, by considering the Tauc relation, the following expressions can be obtained^12^:

6 $$F\left( R \right) \propto {\upalpha } \propto = \frac{{\left( {{\text{hv}} - {\text{Eg}}} \right)^{\frac{1}{n}} }}{{{\text{hv}}}}$$

7 $$F\left( R \right)\left( {hv} \right)^{n} = A\left( {h\nu - Eg} \right)$$

where A is a constant and n is equal to 2 for semiconductors with direct allowed transitions^44^. As shown by the inset graph in Fig. 6b, the value of Eg can be determined by extrapolating the linear part of the function curve with the energy axis. The estimated bandgap energies are 3.10 and 2.96 eV for the Hy-ZnO NPs, and 10% Hy-Co–ZnO NPs respectively.

### X-ray photoelectron spectroscopy (XPS)

**Figure 7:**
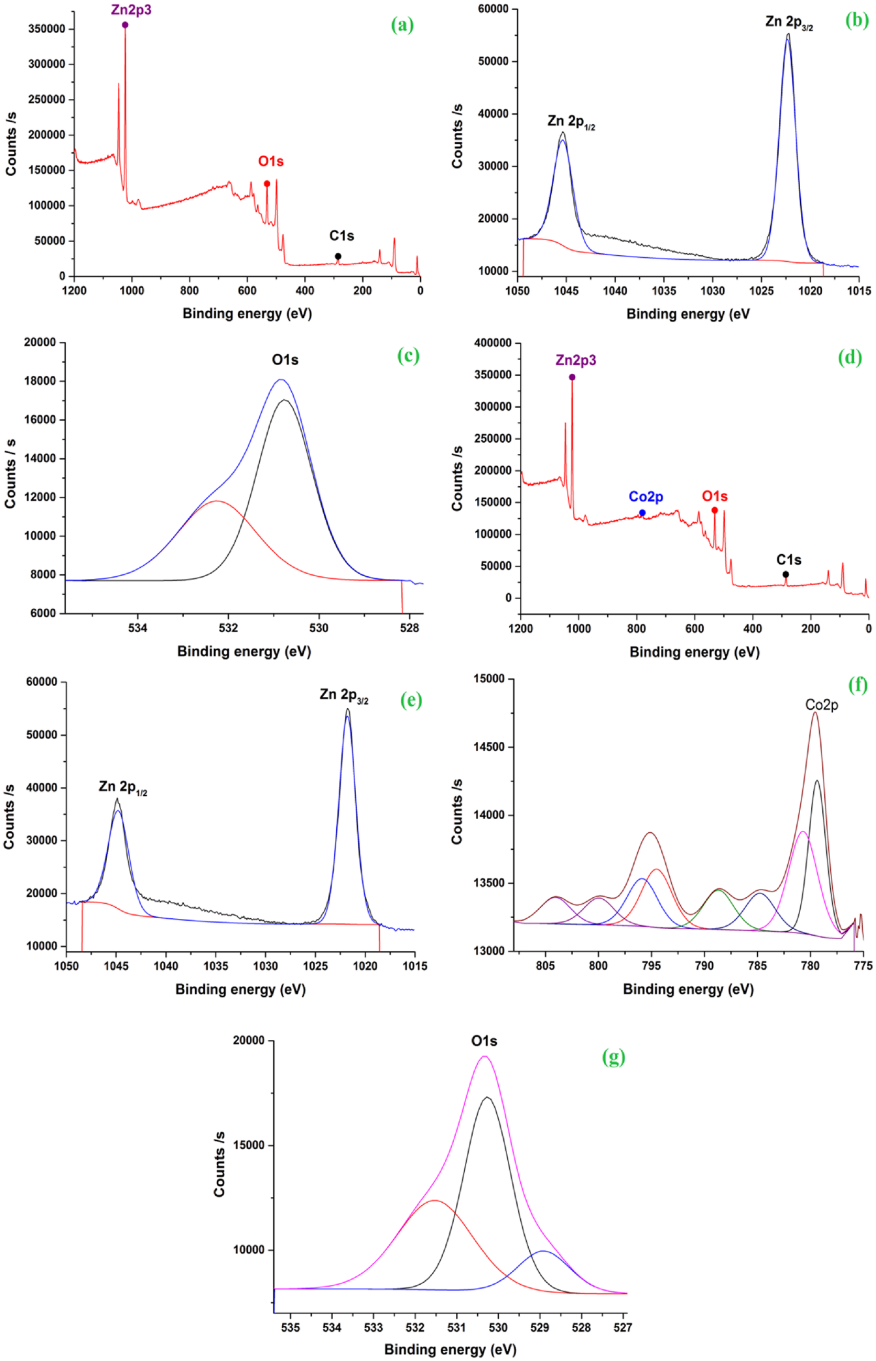
X-ray photoelectron spectra of (**a**–**c**) Hy-ZnO NPs, and (**d**–**g**) 10% Hy-Co–ZnO NPs.

The investigation of the XPS spectra showed that the Co content on the surface of the Hy-Co-doped ZnO is very near to 10% (Fig. 7a–g). In Fig. 7a, XPS investigations provided conclusive proof that Zn is in the 2 + valence state as the binding energy position of the Zn 2p spectra is near to the standard data of zinc oxide^51^. Where Zn 2p_3/2_ and Zn 2p_1/2_ core level binding energies were found to be 1022.34 and 1045.33 eV, respectively (Fig. 7b). The fact that there is a large energy gap (22.9 eV) between these two peaks indicates that Zn is mostly found in the chemical form Zn^2+^^52,53^. Figure 7c shows the bending energy of O1 s in the Hy-ZnO NPs. In Fig. 7d,e, the Zn 2p core level steadily moves to lower binding energy with increasing Co doping, in agreement with Co 2p XPS spectra^51^. In the 10% Co–ZnO NPs that were synthesized, three separate distinctive peaks of the O1s energy state were identified at 528.91, 530.26, and 531.53 eV (Fig. 7g). These peaks are shown in Fig. 7g, and are attributed to the formation of three different types of O. The hexagonal wurtzite structure is anticipated to have zinc and cobalt ions surrounding the lattice oxygen, in accordance with the reduced binding energy. The medium binding energy confirms that the ZnO matrix contains oxygen vacancies. The development of adsorbed oxygen (O^2−^) on the ZnO surface^51^. The Co 2p spectra appear at Co 2p_3/2_ at 779.36 eV and Co 2p_1/2_ at 788.68 eV with satellite peaks shown in the high-resolution Co2p spectra of the sample in Fig. 7f. The divalent state of Co is shown by a 9.32 eV energy difference between Co 2p_3/2_ and Co 2p_1/2_, which is homogeneously bound by oxygen atoms in tetrahedral coordination. The presence of the satellites indicates that the Co ions valence state is 2 + , and Co^2+^ is found within an O^2−^-enclosed tetrahedral crystal field^31^. In the tetrahedral positions of the ZnO lattice, Co^2+^ have thus successfully incorporated lattice.

### Thermal analysis

**Figure 8:**
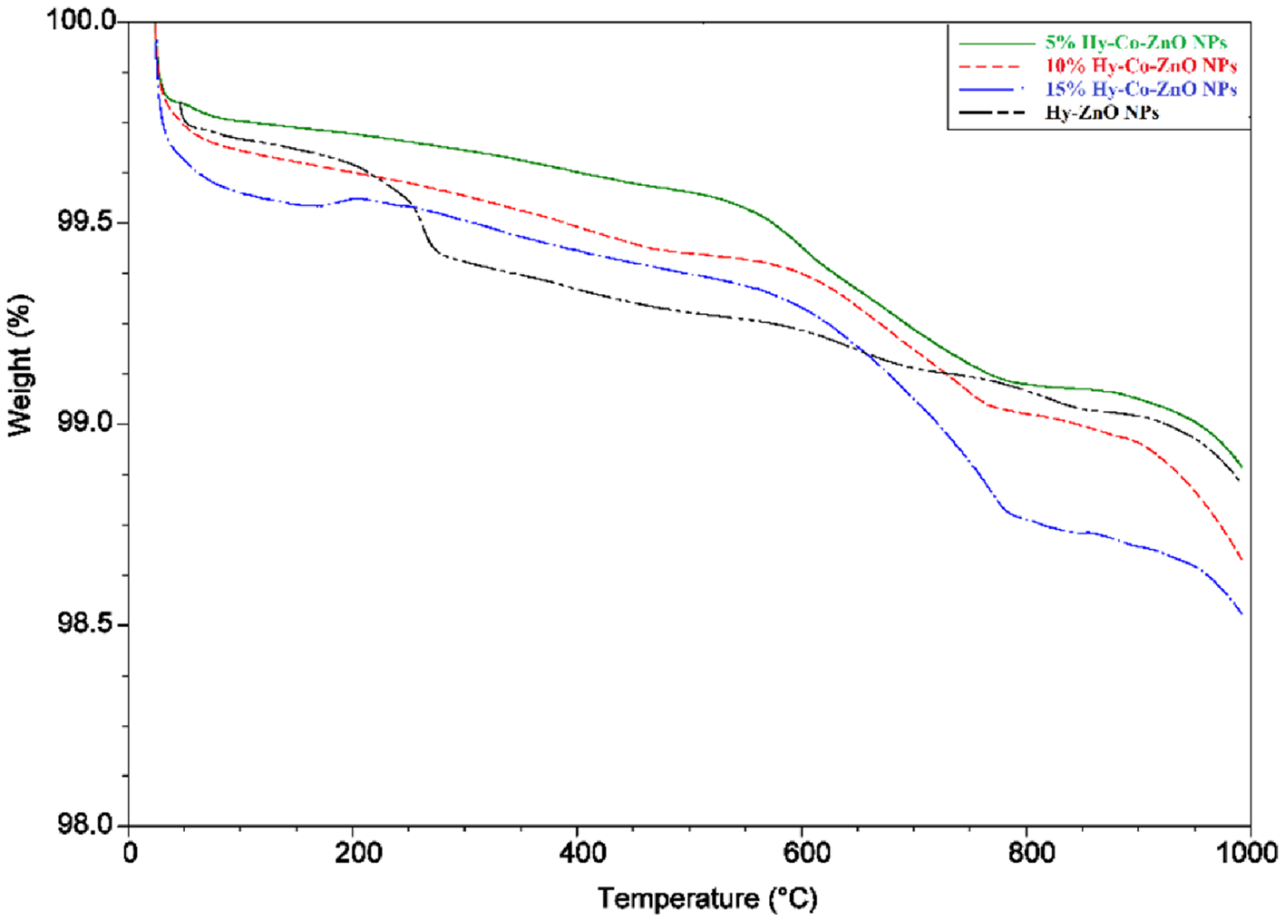
TGA analysis of Hy-ZnO NPs, 5, 10, 15% Hy-Co–ZnO NPs.

TGA measurements were performed to check the thermal stability of nanomaterials. Thermogravimetric (TG) curves of the prepared Hy-ZnO NPs, 5 10, and 15% Hy-Co–ZnO NPs were recorded in Fig. 8 and Figure S3. In the 50–250 °C temperature range, the first weight loss in the four samples was 0.150, 0.269, 0.351, and 0.364%, respectively, which can be attributable to the samples' water content^54^. In the range between 250 and 1000 °C, ZnO experienced six additional weight losses, with the highest loss of mass occurring between 300 and 400 °C (0.243%). Other weight losses for ZnO included 0.102, 0.693, 0.115, 0.0849, and 0.177%. Between 400 and 1000 °C, slightly higher weight losses for the 5% Hy-Co–ZnO, 10% Hy-Co–ZnO, and 15% Hy-Co–ZnO NPs were noted; nonetheless, the generated ZnO had essentially the same thermal stability.

### Photocatalytic studies

#### Photocatalytic test

**Figure 9:**
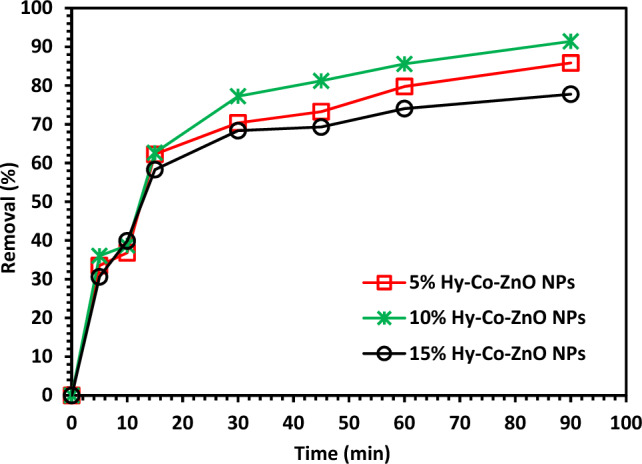
Test of 5, 10, and 15% Hy-Co–ZnO NPs catalyst on CIPF photodegradation.

To determine the best catalyst performance, a specific amount (100 mg) 5, 10, and 15% of Hy-Co–ZnO NPs was added to a 300 mL conical flask containing (100 mL) of CIPF with concentrations of 30 ppm and pH 7 for 2 h under visible light and the removal efficiency was measured. It was shown that 10% Hy-Co–ZnO NPs gave the best performance. So, it has been selected for photocatalytic degradation of CIPF as a model pollutant over 10% Hy-Co–ZnO NPs as a photocatalyst under visible irradiation (Fig. 9).

#### pH point of zero charge (pH_PZC_)

**Figure 10:**
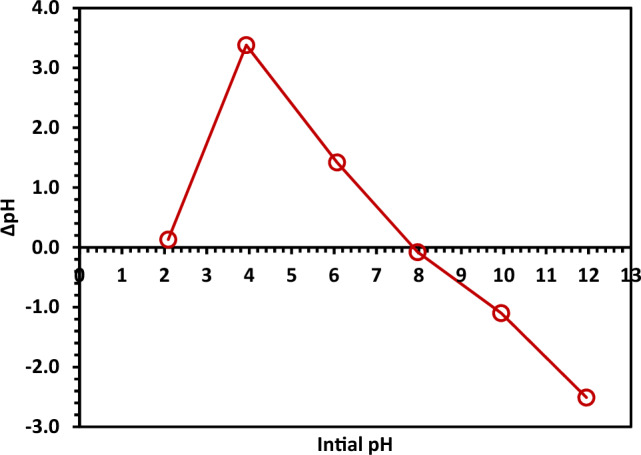
The pH_ZPC_ determination of 10% Hy-Co–ZnO NPs.

The pH drift method was used to determine the pH_PZC_ value for 10% Hy-Co–ZnO NPs using NaCl (0.1 N) as an electrolyte solution^59,60^. Six flasks were filled with 50.0 mL of the 0.1 N NaCl solution, and each was adjusted to pH values (2, 4, 6, 8, 10, and 12) as an initial pH_i_ value. 0.1 g 10% Hy-Co–ZnO NPs was added to each flask and then shaken at 150 rpm for 24 h^59^. The dispersions were then left to settle and the pH was determined and assigned as the final pH_F_ after interaction of the material with the electrolyte solution. A graph was plotted between initial pH and *Δ*pH, where ΔpH = pH_i_ − pH_F_. The pH_ZPC_ value of 10% Co–ZnO NPs was determined by defining the point of intersection^61^, which showed in Fig. 10 to be 8.

#### The impact of pH on CIPF photo-degradation

**Figure 11:**
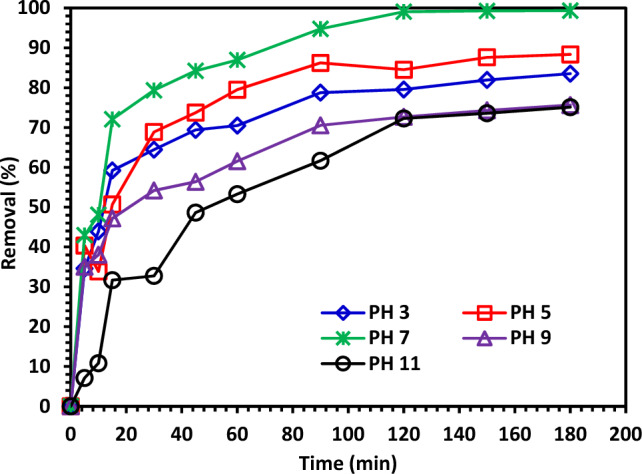
The impact of pH on CIPF (30 ppm) photodegradation in the presence of 10% Hy-Co–ZnO NPs (0.1 g/L).

The solution's pH was changed to 3, 5, 7, 9, and 11 at CIPF = 30 ppm, catalyst dose of 0.1 g/L, temperature of 25 °C, and 200 rpm while being exposed to visible light to evaluate the impact of pH on the photocatalytic degradation of CIP. Figure 11 shows that the neutral pH (7) is the optimal pH for CIPF decomposition using 10% Hy-Co–ZnO NPs. It is difficult to predict how pH would affect the photocatalytic degradation of pollutants because the result mostly depends on the pollutant kind and the photocatalyst pH_ZPC_. The electrostatic interaction between the pollutant molecules and the catalyst surface is significantly influenced by the pH of the solution, which also affects the surface charge of the photocatalyst^55^. Adsorption is necessary for photocatalytic degradation to occur^56^. Because the pH_ZPC_ of the Hy-Co–ZnO NPs is 8, the ZnO surface is positively charged at pH levels below 8 and negatively charged at pH levels above 8^57^. In contrast, CIPF has pka values of 6.09 and 8.2. ZnO and CIPF are both positively charged at acidic pH; as a result, the adsorption on the surface of Hy-Co–ZnO NPs is constrained. Since Hy-Co–ZnO NPs have a positive surface and CIPF has a negative surface at pH values more than 6.09, the antibiotic binds to Hy-Co–ZnO NPs surface and binds there, increasing the rate of destruction. When a solution's pH is greater than 8, CIPF will manifest as an anionic form (CIPF-O), which prevents species from oxidizing and ultimately reduces the effectiveness of CIPF removal^58^.

#### The effect of catalyst dosage on CIPF photodegradation

**Figure 12:**
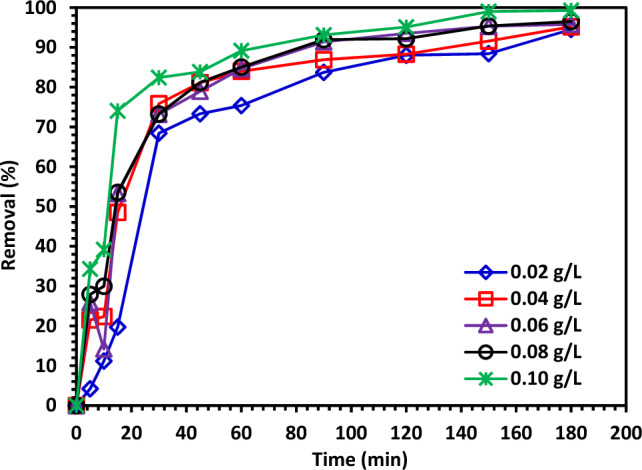
The effect of 10% Hy-Co–ZnO NPs dosage (0.02–0.1 g/L) on CIPF (30 ppm) photodegradation at pH 7 a room temperature.

Testing was done between 20 and 100 mg of photocatalyst to see how the catalyst dose affected the rate of CIPF degradation. The results shown in Fig. 12 showed that 100 mg of 10% Hy-Co–ZnO NPs caused the best decomposition. The findings can be explained by the fact that adding additional catalysts would produce 10% Hy-Co–ZnO NPs centers that were more active and capable of absorbing photons and producing hole-electron pairs^62^. The number of active species, catalyst surface area, and light absorption all increased with increasing catalyst dosage, which accelerated the rate of antibiotic degradation^62,63^. However, at greater dosages outside of the optimal amount, the solid particles can prevent photon penetration; as a result, the overall quantity of photons accessing the catalyst surface to produce radicals was reduced^62^. Additionally, some catalyst components may show up in the dark area and reduce light penetration. When the catalyst concentration is high, other factors that may affect photocatalytic activity include the inactivation of activated molecules through collisions with ground-state molecules, light scattering, screening effects, and the concentration of aggregated nanoparticles^62^.

#### The effect of antibiotic concentration on CIPF photodegradation

**Figure 13:**
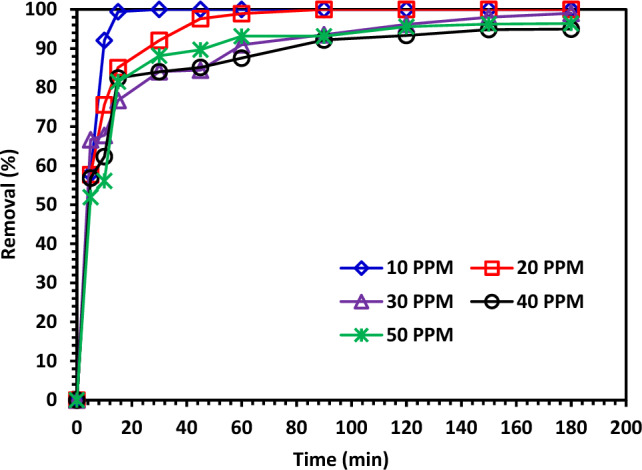
The effect of CIPF concentration (10, 20, 30, 40, and 50 ppm) on its photodegradation in the presence of 10% Hy-Co–ZnO NPs (0.10 g/L).

A crucial variable for optimizing the photocatalytic degradation process is the concentration of antibiotics in the water system. CIPF concentrations of 10, 20, 30, 40, and 50 mg/L were used in the current study. The optimal concentration of 10% Hy-Co–ZnO NPs is shown in Fig. 13 along with the photocatalytic breakdown pattern of CIPF at various concentrations. As CIPF concentration was increased, the photocatalytic activity of the photocatalyst dropped, where the photons cannot reach the catalyst’s surface in the presence of higher antibiotic doses because antibiotic molecules absorb them^64,65^. Additionally, a higher antibiotic concentration favors the antibiotic's adsorption on the surface of the catalyst. These two elements eventually have a diminishing impact on photocatalytic activity at greater concentrations by lowering the yield of exaction and OH radicals^65,66^.

#### The effect of temperature on the improvement of degradation efficiency

**Figure 14:**
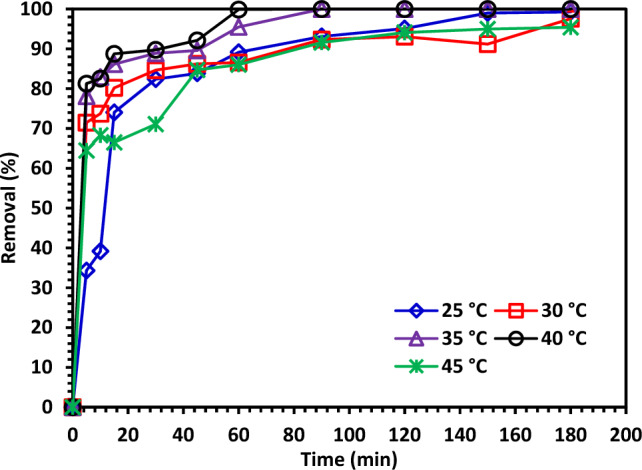
The effect of temperature on degradation efficiency improvement.

Figure 14 shows that the catalytic activity was evaluated to see how temperature affected its photodegradation efficiency. From 25 to 40 °C, the CIPF degradation efficiency increases as the temperature rises due to an increase in catalyst activation^67^. The removal efficiency, however, revealed the minimal degrading efficiency at 45 °C. Higher temperatures cause the radicals to interact with one another rather than the CIPF molecule, which decreases the effectiveness of the degradation process^67^.

#### Effect of shaking speed

**Figure 15:**
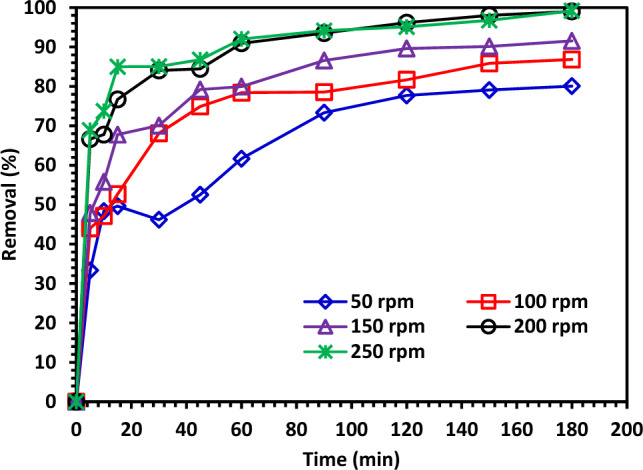
The effect of shaking speed for improvement of CIPF degradation efficiency using 10% Hy-Co–ZnO NPs.

The Continuous shaking enhances solution flow over the surfaces of the photocatalysts and causes a uniform distribution of photocatalysts throughout the solution^68^. The shaking mechanism's impact on the effectiveness of photocatalytic degradation under visible light is depicted in Fig. 15. With increasing shaking speed, the photocatalyst degrading efficiency significantly improved. Shaking increases the effectiveness of CIPF degradation in the aqueous medium by reducing the boundary layer distance, bringing the antibiotic into contact with photocatalysts in the solution, and creating localized turbulence close to the base of the photocatalyst^68^. Degradation efficiency was found to be higher by using 10% Hy-Co–ZnO NPs at 250 rpm.

#### The effect of scavengers on removal efficiency

**Figure 16:**
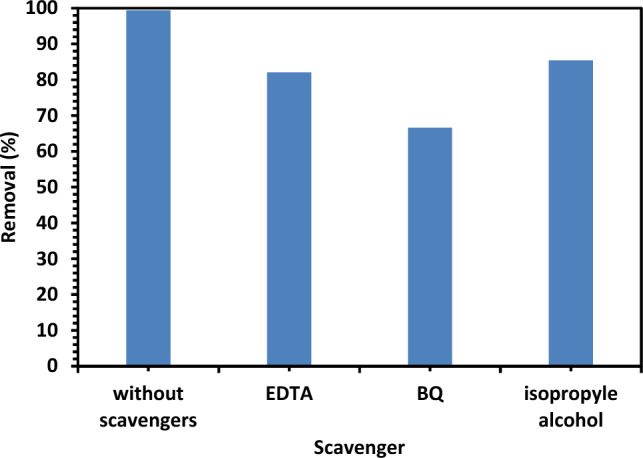
Effect of different scavengers on CIPF photodegradation in the existence of 10% Hy-Co–ZnO NPs as a catalyst (0.10 g/L) at reaction pH = 7.

Trapping experiments have been done using various quenchers, such as isopropyl alcohol (IPA) (10 mM), benzoquinone (BQ) (1 mM), and disodium ethylene diamine tetraacetic acid (2 Na EDTA) (10 mM), which are known to trap HO^·^, O^·2−^ and holes generated in the reaction mixture on excitation of semiconducting material^33^. Figure 16 illustrates the variation in CIPF elimination from water in the presence and absence of several quenchers with 10% Hy-Co–ZnO NPs as photocatalysts. The findings suggest that quenchers such as BQ, EDTA, and IPA have a significant impact on reducing the degradation rate, demonstrating that O_2_ and HO are the primary reactive types in the removal of CIPF.

#### CIPF degradation kinetic study under visible light

**Figure 17:**
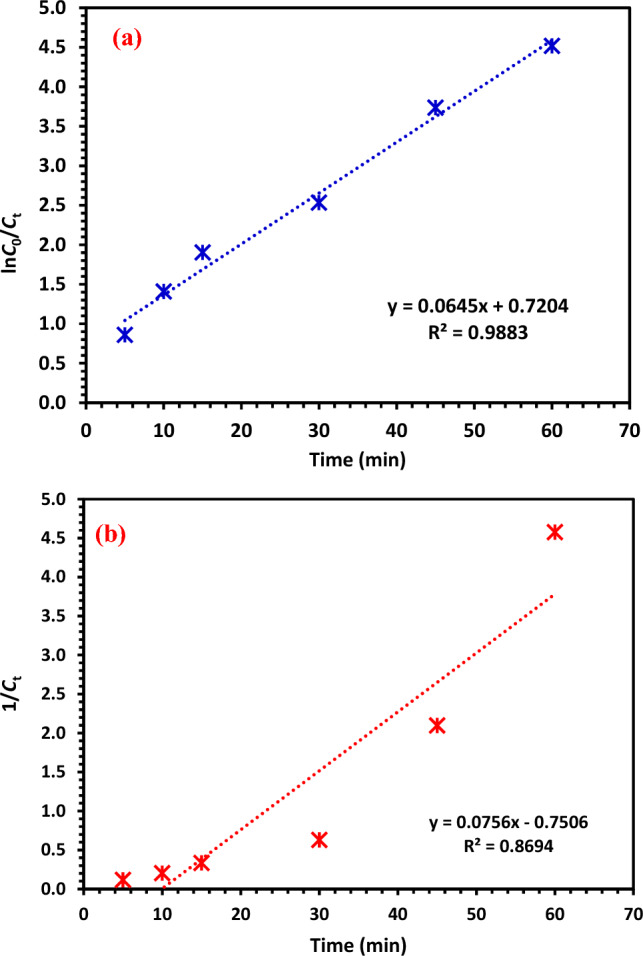
(**a**) First order, (**b**) Second order kinetic study of CIPF degradation under visible light at room temperature.

The kinetics of the CIPF photocatalytic degradation were studied based on Eqs. 8 (first-order) and 9 (second-order). First-order was studied by plotting of ln *C*_t_/*C*_0_ vs. irradiation time (*t)* and the second order was studied by plotting of 1/*C*_t_ vs. time (*t*) (Fig. 17a,b). The ideal conditions (10% Hy-Co–ZnO NPs concentration of 30 mg/L, 180 min irradiation time, and pH value of 7.0) were used for several experiments.

8 $$ln\frac{{C_{0} }}{{C_{t} }} = kt$$

9 $$\frac{1}{{C_{t} }} = K_{2} t + \frac{1}{{C_{0} }}$$

where C_t_, C_0_, and t are the time-dependent CIPF concentration after lamp switching on, CIPF initial concentration, and reaction time, respectively. Also, *k* is stated as the slope of the plot and represents the rate reaction constant^69^. Since the plot in Fig. 17a was linear, it may be assumed that the photocatalytic reaction approximated pseudo-first-order kinetics depending on the *R*^2^ = 0.9883, while the second order in Fig. 17b shows *R*^2^ = 0.8694. As can be seen, the rate constant for CIPF deterioration was 0.0645 min^−1^. The first-order model is suitable for displaying the rate of reaction and amount of CIPF elimination at any given time due to the strong determination coefficient (*R*^2^) and linear relationship between CIPF concentration and exposure time.

#### Recycling of ZnO

**Figure 18:**
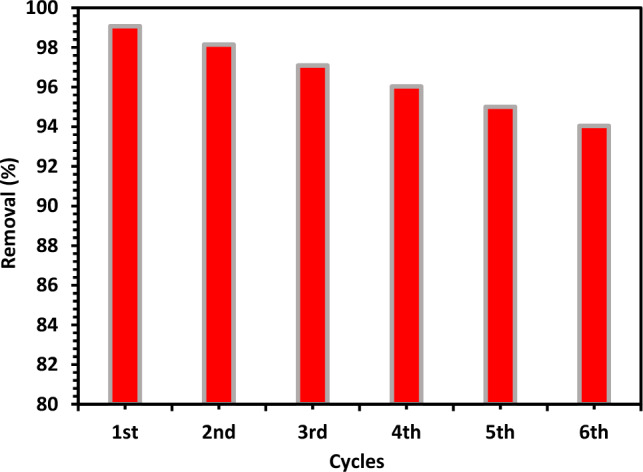
Recyclability of 10% Hy-Co–ZnO NPs within six consecutive cycles for the CIP photodegradation under irradiation with visible light.

For environmentally friendly wastewater treatment, catalyst recycling is crucial. Therefore, it is essential to ascertain whether photocatalysts may be recycled during CIPF photodegradation. To do this, the photocatalyst was reused Six times after a thorough wash with ethanol and distilled water every time for photodegradation of CIPF. Photocatalyst was observed to have almost the same photocatalytic activity in each cycle. Figure 18 led to only a slight low in the catalyst photocatalytic activity, indicating the enhanced photocatalytic stability of the material.

#### Mechanism of photocatalytic activity

**Figure 19:**
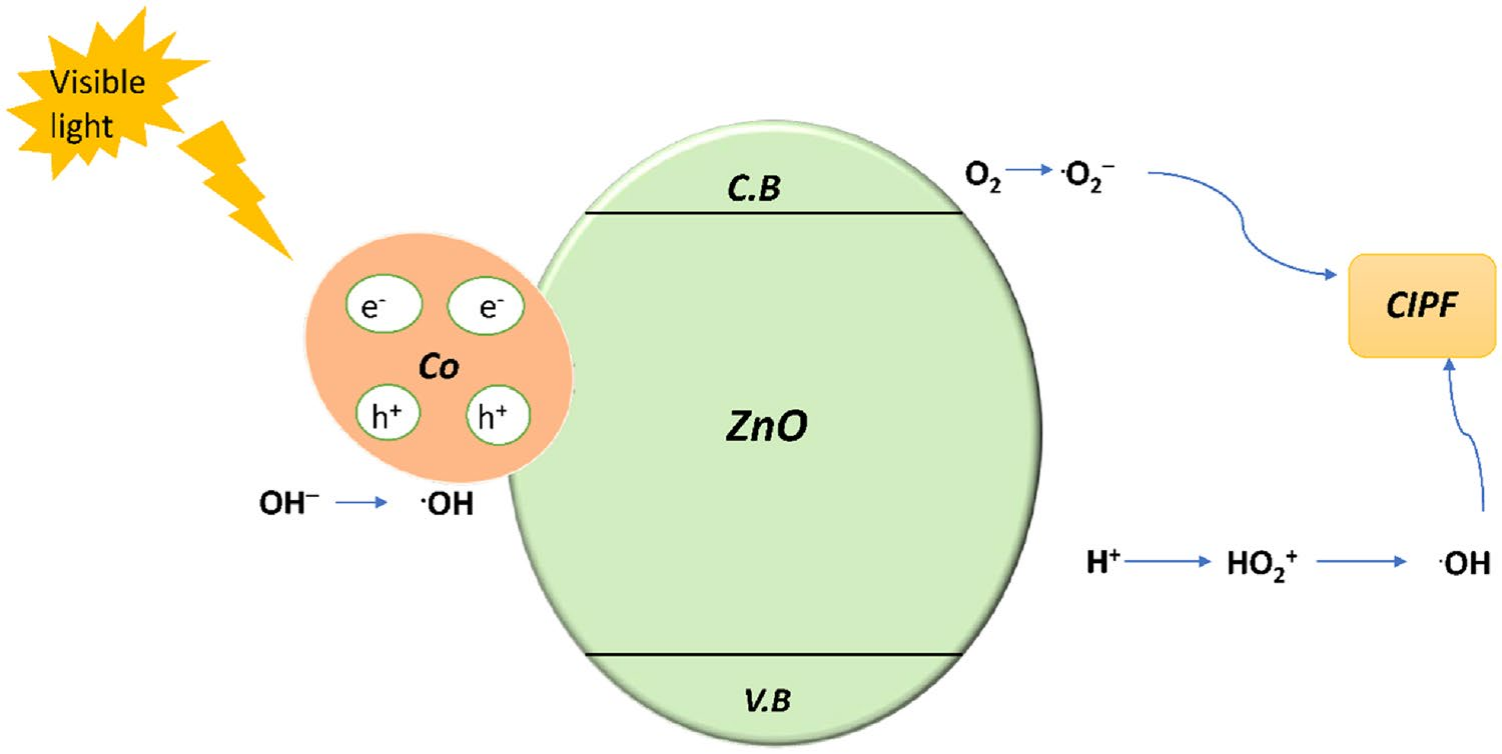
A photocatalytic mechanism diagram of Co^2+^-doped ZnO NPs sample under visible light excitation.

The main element affecting the photocatalytic activity of the Co^2+^ doped ZnO sample is the doping concentration of Co^2+^ ions. It is widely accepted that the production of photogenerated e–h° pairs during excitation and the confinement of the excitations are intimately related to each other and photocatalytic activity in semiconductor oxide^70,71^. In Fig. 19, a possible method is presented for creating photogenerated e–h° pairs in Co2 + doped ZnO nanoparticles under visible light. As seen in Eq. (10), the formation of the e–h° pairs occurs in the first step when they are activated by visible light. Then, Eq. (11) demonstrates that superoxide anion radicals (O^2·−^) will be created when the electrons produced in the excited state of Co^2+^ contact with adsorbed oxygen molecules on the ZnO surface. While the ZnO valence band holes will interact with hydroxyl groups to create highly reactive hydroxyl radicals (^**.**^OH), as shown in Eq. (12), they can also interact with H_2_O molecules, as shown in Eq. (13). In conclusion, the interaction between antibiotics and superoxide (O^2·−^) or hydroxyl radicals (^**.**^OH), as indicated in Eqs. (14), and (15), is required for the breakdown of antibiotics^70^.

10 $${\text{ZnO}}:{\text{Co}}^{2 + } + {\text{h}}\nu \to {\text{e}}^{-} + {\text{h}}^{ + }$$

11 $${\text{e}}^{-} + {\text{O}}_{{2}} \to {}^{ \cdot }{\text{O}}_{2}^{ - }$$

12 $${\text{h}}^{ + } + {\text{OH}}^{-} \to {\text{ OH}}^{ \cdot }$$

13 $${\text{h}}^{ + } + {\text{H}}_{{2}} {\text{O}} \to {\text{H}}^{ + } + {\text{OH}} \cdot$$

14 $${\text{OH}}^{ \cdot } + {\text{CIPF}} \to {\text{degradation}}\;{\text{products}}$$

15 $$\cdot {\text{O}}^{{{2}{-}}} + {\text{CIPF}} \to {\text{degradation}}\;{\text{products}}$$

The band-gap Narrowing of Co^2+^ doped ZnO resulting from the introduction of substituent Co^2+^ ions into the ZnO lattice could hinder e–h° pair recombination when the dopant level of Co^2+^ ions is between 5 and 15 mol%^71^. As a result, the concentration of holes will rise, improving the photocatalysts' efficiency. With further boosting the Co^2+^ ions concentration (15 mol%), however, there is a little decline in the photocatalytic effectiveness, which may be caused by competition between the substitution and interstitial interaction of dopant ions. In addition to taking the place of Zn^2+^ ions in samples with high Co^2+^ ion concentrations, Co^2+^ ions may also settle at interstitial regions where they act as traps or sites for the recombination of holes and photoexcited electrons^71^. The obtained photocatalytic degradation OF Co–ZnO photocatalyst results were compared with earlier related works in Table S3.

#### CCD optimization results

The recovery of the CIPF antibiotic was examined using a four-factor central composite design (CCD), which was studied based on 30 experimental runs at different numerical values of photocatalyst dosage (A), primary CIPF solution concentration (B), reaction speed (C), and pH (D). Design expert version 13.0.5.0 was used to examine the response. Table 4 displays the independent factors’ coded and uncoded values according to the 30 experiments’ CCD along with their responses. Equation (16) describes the quadratic model in terms of coded factors.

16 $$\begin{aligned} {\text{Removal}}\% & = 82.36 + 0.338{\text{A}} + 11.48{\text{B}} + 6.52{\text{C}} + 6.02{\text{D}} + 0.51{\text{AB}} + 1.64{\text{AC}} + 2.68{\text{AD}} \\ & \quad - 1.14{\text{BC}} + 0.976{\text{BD}}{-}3.49{\text{CD}} + 0.438{\text{A}}^{2} {-}4.27{\text{B}}^{2} {-}1.71{\text{C}}^{2} {-}5.49{\text{D}}^{2} \\ \end{aligned}$$

**Table 4:** Results of CCD experiment design with experimental and expected values for CIPF's photodegradation performance.

Run	A Catalyst dosage	B Antibiotic dosage	C Shaking speed	D PH	Actual value	Response in % CIPF removal Predicted value
1	60	30	250	7	91.6584	88.57
2	100	30	150	7	91.526	84.79
3	40	40	200	9	94.0417	89.54
4	60	30	150	7	82.3568	82.36
5	80	20	200	5	68.3217	67.77
6	80	40	100	5	52.135	60.44
7	80	20	200	9	69.1658	76.24
8	80	20	100	5	41.2114	42.18
9	60	30	150	11	74.0814	72.45
10	60	30	150	7	82.3568	82.36
11	60	30	150	7	82.3568	82.36
12	80	40	200	9	90.8391	93.85
13	40	20	200	5	69.0168	64.15
14	40	40	100	5	84.0367	73.44
15	40	40	100	9	81.0576	89.05
16	60	30	150	7	82.3568	82.36
17	40	40	200	5	77.4081	87.90
18	60	50	150	7	94.4588	88.24
19	60	30	150	7	82.3568	82.36
20	20	30	150	7	80.6024	83.43
21	60	30	50	7	63.3234	62.50
22	80	20	100	9	67.6762	64.62
23	60	30	150	7	82.3568	82.36
24	60	10	150	7	40.0198	42.33
25	40	20	100	9	60.6752	56.85
26	40	20	100	5	40.7149	45.14
27	80	40	100	9	85.4518	86.79
28	80	40	200	5	81.1817	81.48
29	60	30	150	3	50.6454	48.36
30	40	20	200	9	62.7606	61.89

Using 10% Hy-Co–ZnO NPs, Fig. S4 illustrates the link between normal, expected, and residual actual charts connected to the experimental data in the CIPF degradation performance. The scattering of data may be seen as a straight line in Figs. S4a and S4b, indicating that the experimental results were influenced by the response’s expected values. Additionally, the photodegradation efficiency of CIPF was shown by the curves for residuals vs run number and residuals versus anticipated quantity in Figs. S4c and S4d, respectively. As shown in Fig. S4c, the residuals versus run number plot displays random scattering at zero with a change of 4.0. This result demonstrates how evenly distributed the data were throughout the model responses^72,73^. Additionally, Figure S4d demonstrates that there is no specific form to the discrepancy between experimental and predicted residues. As a result, it can be said that the produced residues have the typical scattering and that the suggested model is adequate^74^. The Box-Cox plot for power transforms, which can be shown in Fig. S5a, indicates that the response of the photodegradation efficiency of CIPF shows that no transformation is indicated since lambda is almost 1. The absence of effect points in our model is confirmed by Cook's distance values less than or equal to 0.50 (Fig. S5b)^73,75^.

The performance of the CIPF's photodegradation was studied using linear, two-factor (2F), quadratic, and cubic models to choose a model and regression equations. The compatibility of each model was examined, and the results are shown in Table S4. Both quadratic and linear models are suggested as suitable models in the current work to anticipate the photodegradation performance of CIPF because they have a higher *F*-value than cubic and 2FI models. Out of these two models, the quadratic model is chosen as the most appropriate for the current inquiry.

Table S5 displays the analysis of variance that matched the experimental outcomes. Model significance is suggested by the model's F-value, which is 11.73. Only 0.01% of noise could account for an F-value this high. Additionally, when the *P*-value is less than 0.0500, model terms are considered significant^75^. Table S5 indicates obviously that the model has a high *R*^2^ value of 0.9163 for removing the CIPF. This indicates that the Predicted *R*^2^ of 0.5179 is not as close to the Adjusted *R*^2^ of 0.8382 as one may generally predict; in other words, the difference is higher than 0.2. This can indicate a major block effect or point to a problem with your model or data. The signal-to-noise ratio is used to gauge the model's adequate accuracy. It is recommended that the ratio be greater than 4. The ratio of 11.354 indicates a suitable signal. This form can be applied to study the design space. The ratio of standard deviation to average mean is used to calculate the coefficient of variation (C.V.), which is a measure of relative variability. The dispersion level around the mean is bigger and this optimization study is less reliable the higher the C.V 8.75%. Using Eq. (13), it is shown that there are a total of four linear effects, six mutual effects, and four second-rate effects that interact to determine the relationship between CIPF removal efficiency and operational parameters. The antibiotic dosage that had the highest positive coefficient in the equation was the factor that contributed the most to the elimination of CIPF. The perturbation graph for the CIPF removal settings is shown in Fig. S6. According to this graph, it is possible to compare the impacts of different factors on the photodegradation response by holding variables constant at specific design space locations when using the RSM approach. Each parameter's steep slope or curve in this graph illustrates how susceptible the reaction is to that particular parameter.

**Figure 20:**
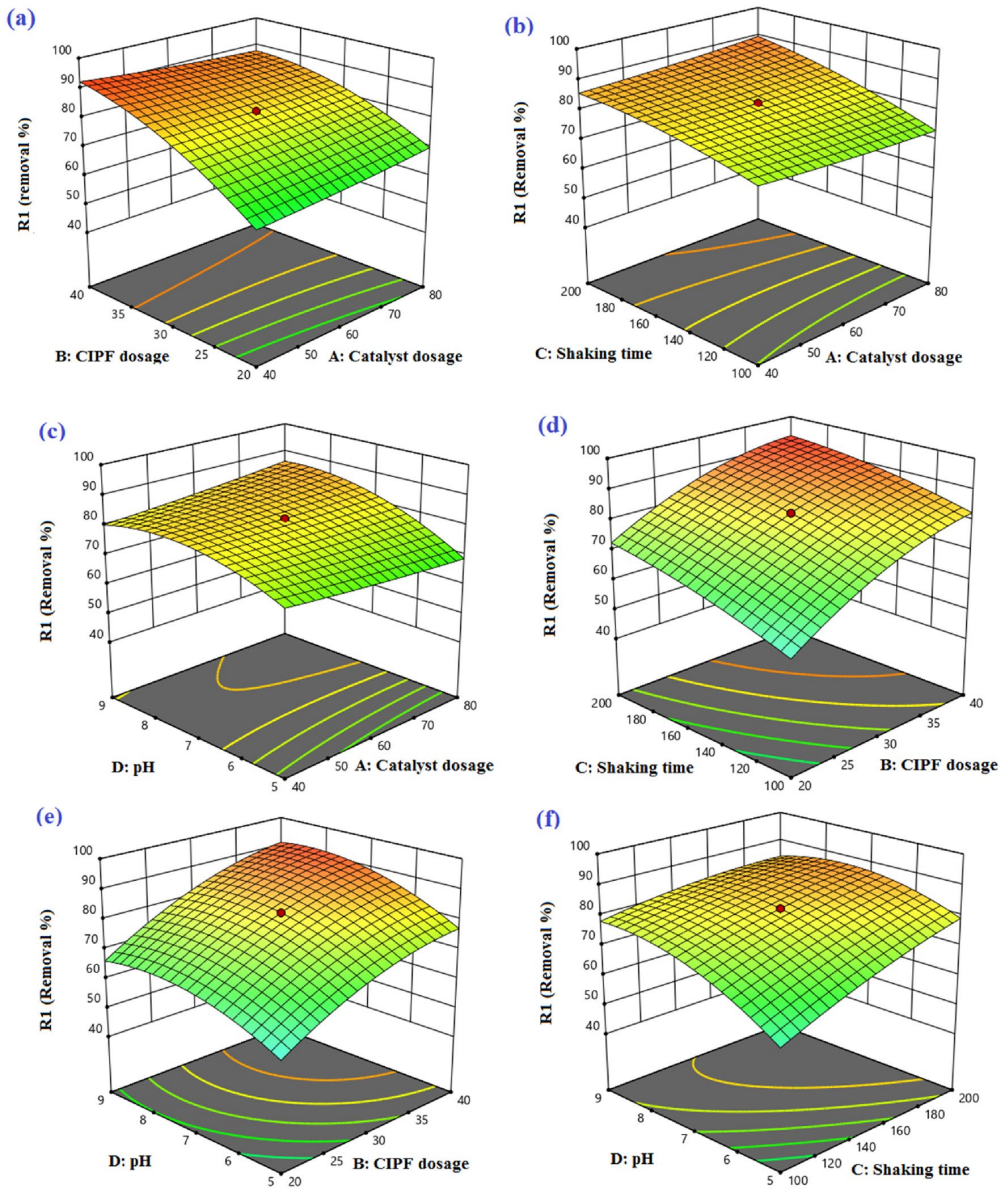
Plots show the photodegradation effectiveness of CIPF in three dimensions.

In the current study, the regression equation utilized to find the optimal quantities of various components was graphically represented by the 3D surface response plots and 2D contour plots. These plots are often used to gain a complete grasp of how the various factors interact with one another in the response. Figure 20 and Figure S7 show the results of the interactions between the four separate components, the 3D surface response, and the 2D contour plots. Figure 20a illustrates the interaction effect of the photocatalyst dosage amount and the concentration of CIPF on the degradation of CIPF while maintaining the optimal values of 150 rpm for the shaking rate and 7 for the pH. The results showed that raising photocatalyst dosage quantity results in an increase in CIPF degradation. In Fig. 20b, increasing the catalyst dosage and shaking rate enhances the photodegradation efficiency of CIPF. According to Fig. 20c, the interaction between photocatalyst dose and pH value on the CIPF degradation efficiency demonstrates that increasing photocatalyst dosage and raising pH from 5 to 7 before lowering at 9 improved photodegradation. Figure 20d shows that with increasing shaking rate and antibiotic dosage amount, the rate of photodegradation also rises. By raising the CIPF concentration and pH value, as presented in Fig. 20e, the photodegradation efficiency of CIPF increases. The interaction between pH and shaking rate on the CIPF's degradation efficiency reveals that raising both the pH and shaking rate increased photodegradation (Fig. 20f).

**Figure 21:**
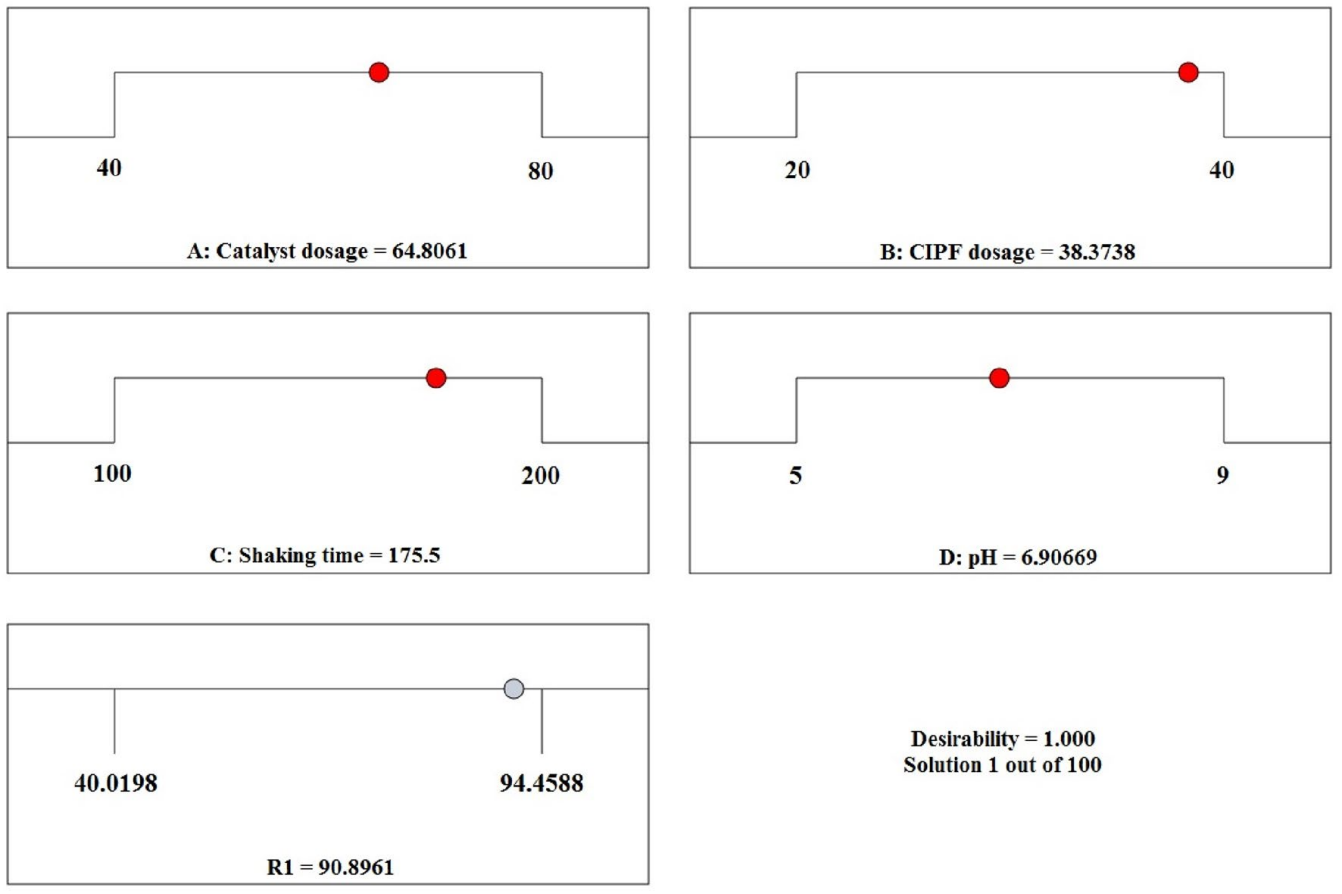
Optimized values for a final response via CCD.

With the use of Design Expert software and the RSM model, the ideal values of several variables, including pH, CIPF concentration, shaking rate, and photocatalyst dosage, were projected for numerical optimization. The greatest CIPF deterioration efficiency was set as the ideal goal in the RSM model. Figure 21 illustrates the optimal value of each parameter and desirability function. According to Fig. 21, at pH 6.91, 64.81 mg of photocatalyst, 38.37 ppm of CIPF, and 175.5 rpm of shaking, the maximal photodegradation rate of CIPF is approximately 94.46%. The desirability function of 1 showed the ideal circumstances for 10% Hy-Co–ZnO NPs to act as a photocatalyst in the photodegradation of CIPF. The desirability of 1 demonstrates the model's acceptability and applicability, demonstrating that the RSM model is a useful tool for creating ideal conditions. Additionally, it showed a strong correlation with the expected outcome, supporting the model's suitability and validity.

## Conclusions

The existing study determines the fabrication and characterization of ZnO and 5, 10 and 15% Co–ZnO for the CIPF photodegradation. The establishment of Hy-Co–ZnO NPs was proved by FTIR, XRD, SEM, EDX, XPS, TGA, and DR-UV–vis spectroscopy. The photocatalytic CIPF degradation indicated that 10% Hy-Co–ZnO NPs significantly enhanced the photocatalytic activity with high removal reaching 98% after 60 min. The impact of each experimental parameter including recycling of the 10% Hy-Co–ZnO NPs catalyst, 10% Hy-Co–ZnO NPs dosage, pH, temperature, and CIPF initial concentrations on photocatalytic activity was investigated. The kinetics studies were also achieved and the outcomes revealed that the first-order kinetic is the suitable model to describe the photodegradation of CIPF. Moreover, CCD optimization of the 10% Hy-Co–ZnO NPs was also studied, and the result revealed that at 10% Hy-Co–ZnO NPs dosage of 64.81 mg, 38.37 mg/L of CIPF primary concentration, pH 6.91, and 175.5 rpm, the removal can reach 90.9% after 180 min.

### Supplementary Information

Supplementary Information.

## Data Availability

The datasets used in this investigation are accessible for review upon request from the paper's corresponding author.
