# Supplementary material for: Hydrothermal fabrication, characterization and RSM optimization of cobalt-doped zinc oxide nanoparticles for antibiotic photodegradation under visible light
Source: Sci Rep. 2024 Jan 23;14:2016. doi: 10.1038/s41598-024-52430-8 (PMC11231344; doi:10.1038/s41598-024-52430-8)
Supplement: Supplementary file 1 — Supplementary Information. [file 41598_2024_52430_MOESM1_ESM.docx]

Supplementary data

**Hydrothermal fabrication, characterization and RSM optimization of cobalt-doped zinc oxide nanoparticles for antibiotic photodegradation under visible light**

Asmaa I. Meky^1^, Mohamed A. Hassaan^2^, Howida A. Fetouh^1^, Amel M. Ismail^1^, Ahmed El Nemr^2^*

**Table S1**. Analysis of the surface area of ZnO NPs and Co doped ZnO NPs.

| Model | Parameter | Hy-ZnO NPs | Hy-Co-ZnO NPs | | |
| --- | --- | --- | --- | --- | --- |
|  |  |  | 5% | 10% | 15% |
| BET | *a*_s,BET_ (m^2^∕g) | 8.3923 | 4.9759 | 10.053 | 9.6253 |
|  | *V*_m_ (cm^3^ STP/g) | 1.9282 | 1.1432 | 2.3098 | 2.2115 |
|  | Mean pore diameter *P*_m_ (nm) | 10.896 | 13.922 | 12.107 | 11.686 |
|  | Volume of total pore *V*_T_ (cm^3^/g) | 2.2861E-02 | 1.7318E-02 | 3.0428E-02 | 2.8120E-02 |
| BJH ads | *V*_p_ (cm^3^/g) | 2.2947E-02 | 1.7406E-02 | 3.0277E-02 | 2.8232E-02 |
|  | *a*_p_ (cm^3^/g) | 8.5805 | 5.3244 | 10.351 | 10.328 |

| 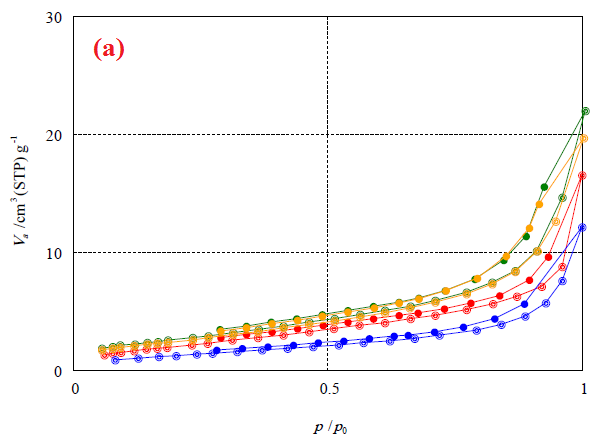 | 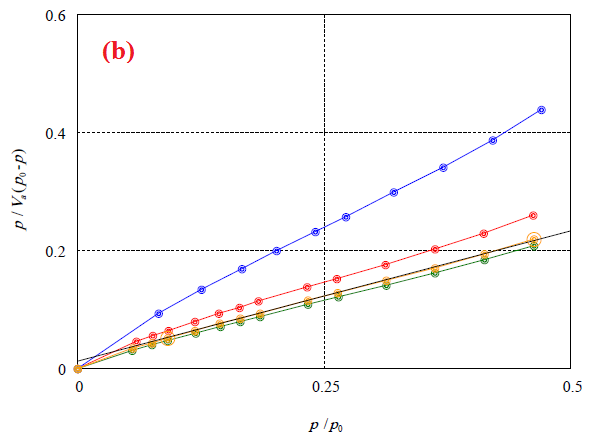 |
| --- | --- |
| 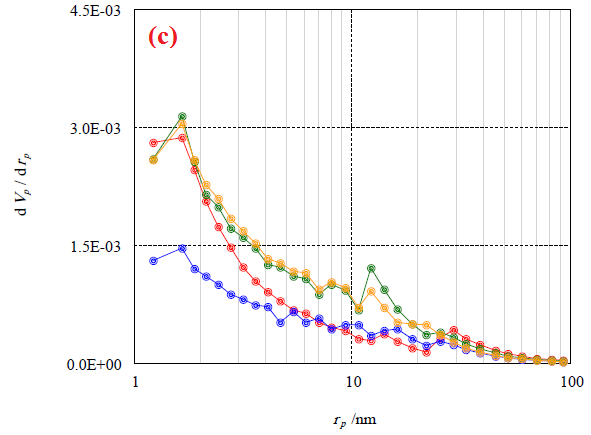 | |

**Figure S1**. (**a**) Adsorption–desorption of Hy-ZnO NPs (red), 5% Hy-Co-ZnO NPs (blue), 10% Hy-Co-ZnO NPs (green) and 15% Hy-Co-ZnO NPs (yellow); (**b**) BET analysis; (**c**) BJH analysis using adsorption isotherm.


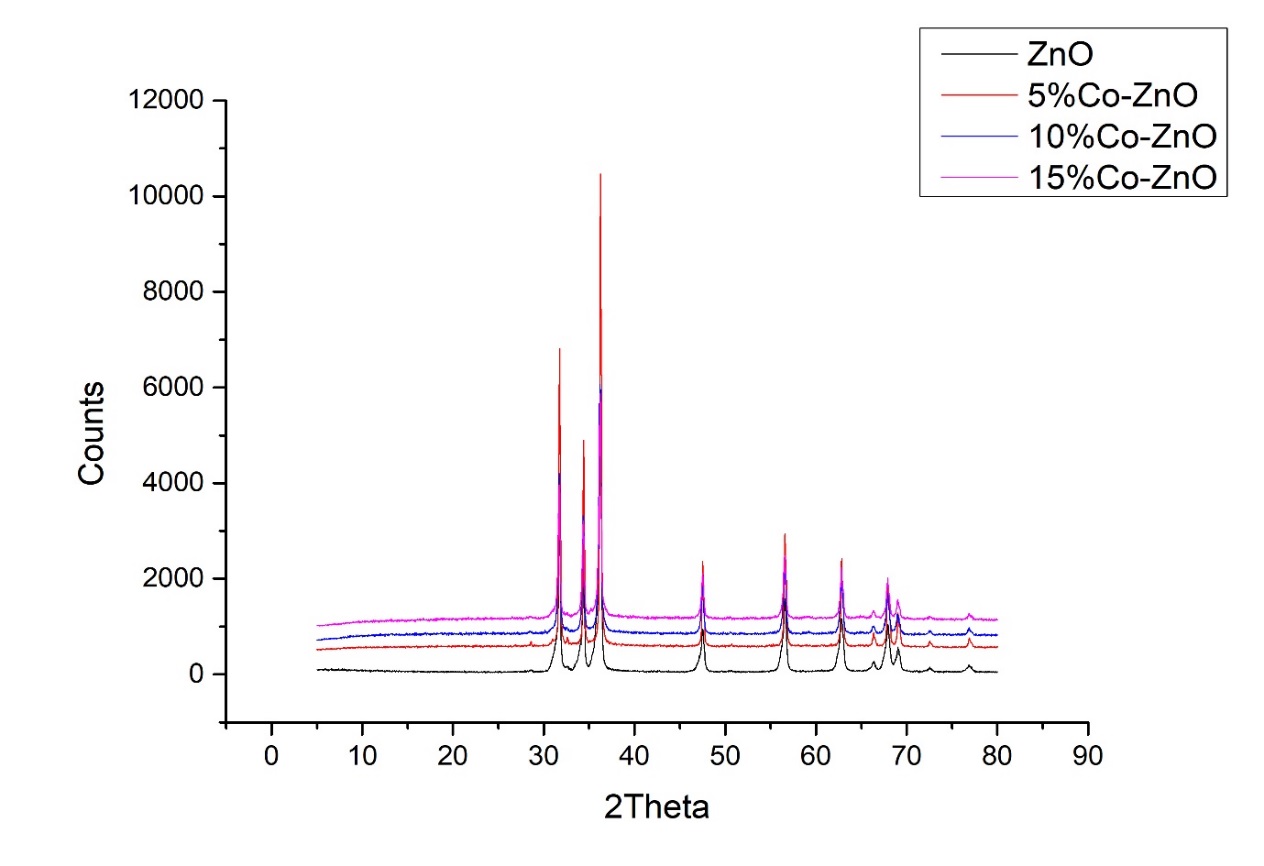


**Figure S2**. X-ray diffraction pattern of ZnO, 5% of Co doped ZnO, 10% of Co doped ZnO and 15 % of Co doped ZnO**.**

**Table S2**. Crystal size of Hy ZnO NPs and 5, 10, 15% Hy-Co-ZnO NPs (nm).

| **2Theta** | **ZnO-NPs** | **2Theta** | **5%Co-ZnO** | **2Theta** | **10%Co-ZnO** | **2Theta** | **15% Co-ZnO** |
| --- | --- | --- | --- | --- | --- | --- | --- |
| 31.75 | 57.29 | 31.754 | 76.20 | 31.718 | 54.50 | 31.717 | 52.67 |
| 34.41 | 61.26 | 34.403 | 79.02 | 34.368 | 55.26 | 34.374 | 57.26 |
| 36.24 | 51.58 | 36.237 | 73.54 | 36.198 | 49.95 | 36.2 | 54.40 |
| 47.52 | 40.43 | 47.526 | 64.44 | 47.488 | 47.95 | 47.506 | 56.49 |
| 56.57 | 43.29 | 56.576 | 59.53 | 56.531 | 47.62 | 56.54 | 51.63 |
| 62.83 | 35.10 | 62.835 | 59.37 | 62.808 | 49.14 | 62.814 | 51.72 |
| 66.34 | 46.49 | 66.353 | 63.07 | 66.358 | 52.44 | 66.33 | 50.95 |
| 67.93 | 40.10 | 67.92 | 58.34 | 67.882 | 47.39 | 67.907 | 51.71 |
| 69.05 | 42.82 | 69.062 | 57.27 | 69.01 | 50.89 | 69.019 | 52.35 |
| 72.54 | 54.44 | 72.556 | 67.86 | 72.564 | 69.37 | 72.542 | 66.41 |
| 76.88 | 33.94 | 76.913 | 81.69 | 76.882 | 57.71 | 76.899 | 56.37 |

| 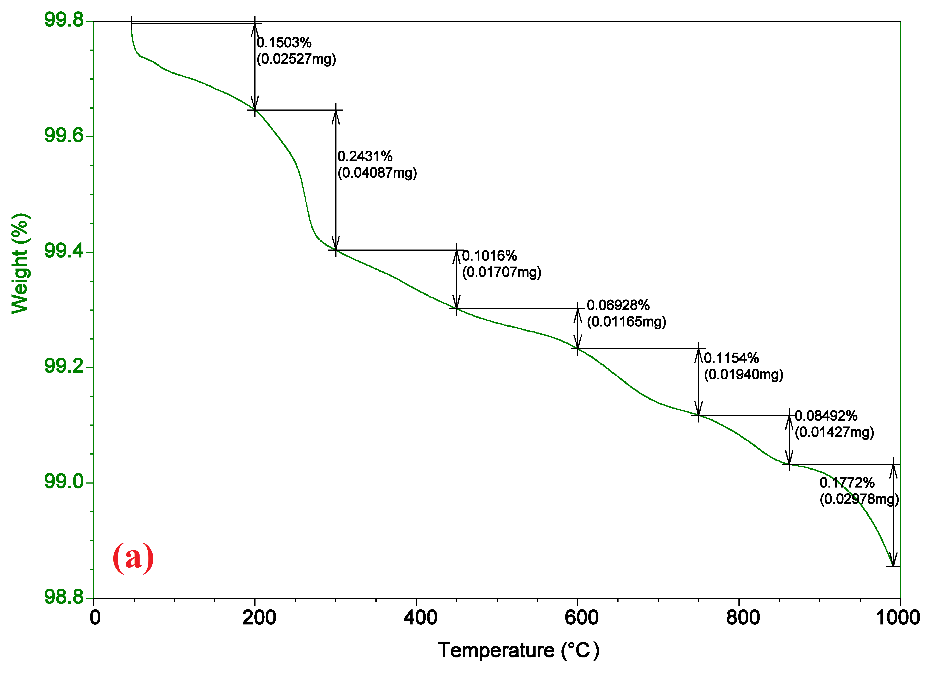 |
| --- |
| 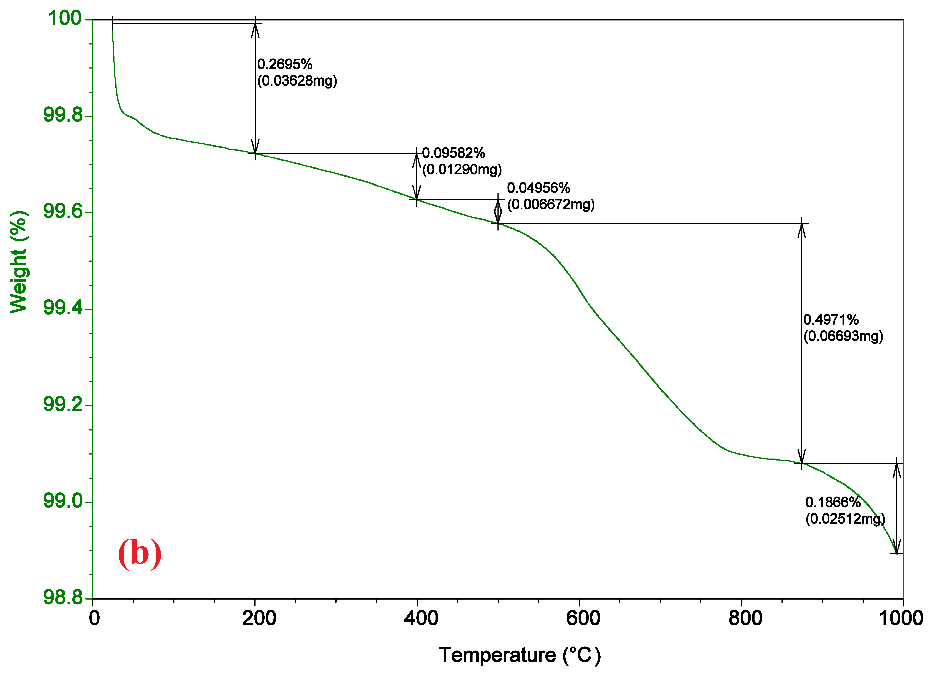 |
| 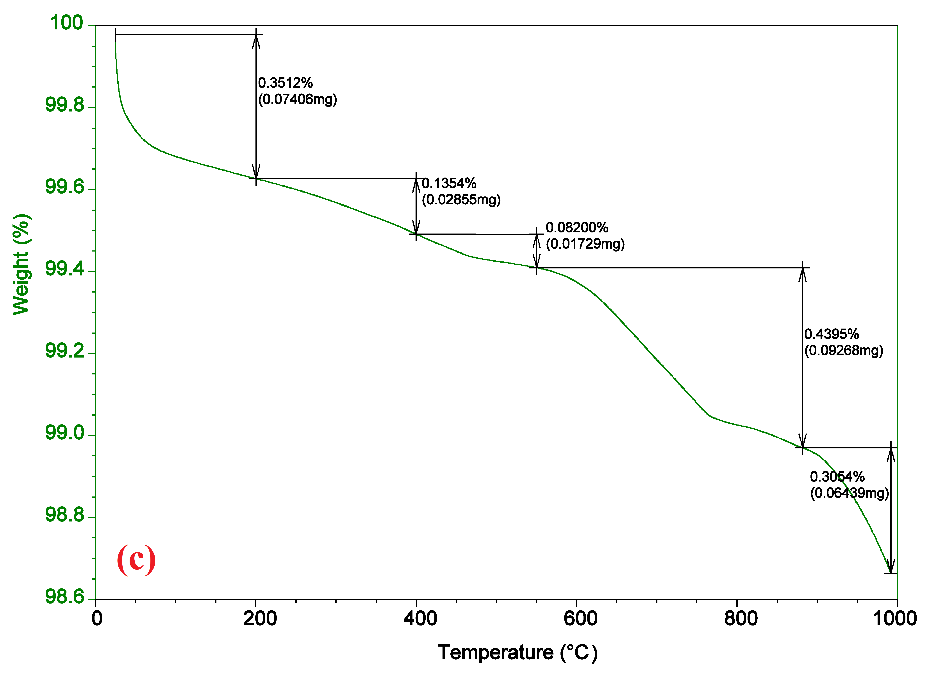 |
| 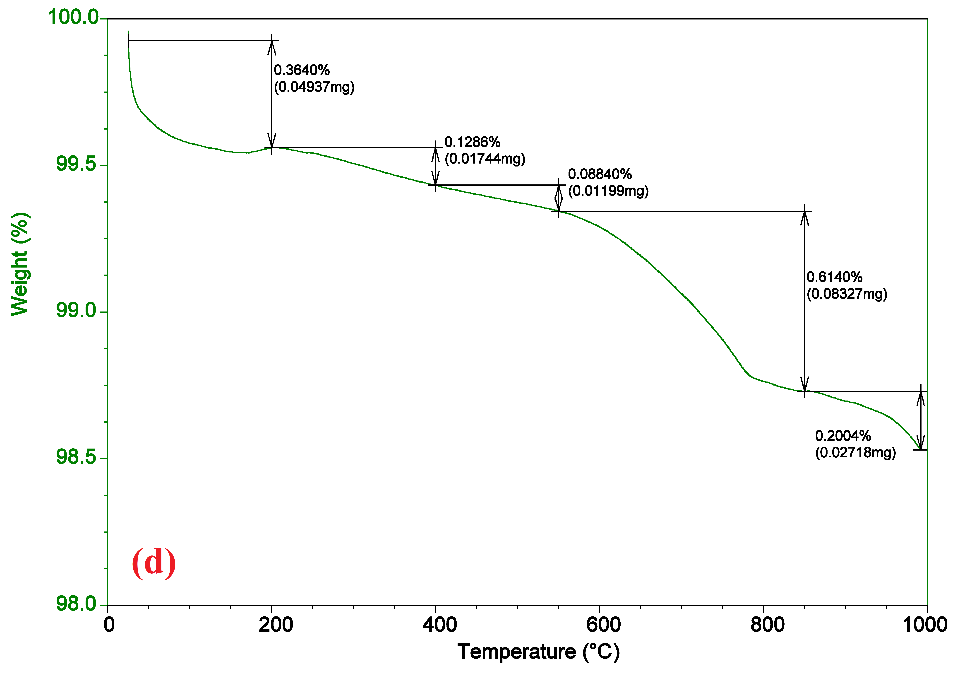 |

**Figure S3**. TGA analyses of (a) Hy-ZnO NPs, (b) 5% Hy-Co-ZnO NPs, (c) 10% Hy-Co-ZnO NPs, and (d) 15% Hy-Co-ZnO NPs.

**Table S3**. Comparison of the present photocatalytic performance with other different literatures work.

| **Photocatalyst** | **Light source** | **Conditions** | **Degradation efficiency (%)** | **Ref.** |
| --- | --- | --- | --- | --- |
| ZnO: Co (5%) | Under visible light | methylene blue (MB), after 300min | 65 | [1] |
| ZnO: Co nanorods | Under artificial solar spectrum | Rhodamine-B (RhB), after 100min | 66.5 | [2] |
| ZnO: Co (4%) | 16W UV lamp with a wavelength of 254 nm | Congo Red, after 120min | 54.1 | [3] |
| ZnO: Co (4%) thin film | 16W UV lamp with a wavelength of 254 nm | methylene blue (MB), after 100min | 76.31 | [4] |
| ZnO: Co (3%) thin films | 23-W xenon lamps with a cut-off filter of 420 nm were used as the visible light source | methylene blue (MB), 120min | 92 | [5] |
| ZnO: Co (5%) nanodisks | under sunlight irradiation | methylene blue (MB), 8min | 95 | [6] |
| Co-doped ZnO NPs | under sunlight | Rhodamine B | 81 | [7] |
| ZnO: Co (10%) | visible-LED-light source (wavelength range from 405 to 800 nm) | 20 mg/L of CIPF solution was attained within 60 minutes | >98 | This work |

**Table S4**. A summary of the tested statistical method for CIPF degradation.

| **Source** | **Sum of Squares** | **df** | **Mean Square** | **F-value** | **p-value** |  |
| --- | --- | --- | --- | --- | --- | --- |
| Mean vs Total | 1.622E+05 | 1 | 1.622E+05 |  |  |  |
| **Linear vs Mean** | **5054.95** | **4** | **1263.74** | **13.34** | **< 0.0001** | **Suggested** |
| 2FI vs Linear | 490.21 | 6 | 81.70 | 0.8265 | 0.5637 |  |
| **Quadratic vs 2FI** | **1256.90** | **4** | **314.22** | **7.59** | **0.0015** | **Suggested** |
| Cubic vs Quadratic | 455.10 | 8 | 56.89 | 2.40 | 0.1332 | Aliased |
| Residual | 166.17 | 7 | 23.74 |  |  |  |
| Total | 1.697E+05 | 30 | 5655.33 |  |  |  |

**Table S5**. Model Summary Statistics.

| **Source** | **Sum of Squares** | **df** | **Mean Square** | **F-value** | **p-value** |  |
| --- | --- | --- | --- | --- | --- | --- |
| **Model** | 6802.05 | 14 | 485.86 | 11.73 | < 0.0001 | significant |
| A-Catalyst dosage | 2.75 | 1 | 2.75 | 0.0663 | 0.8003 |  |
| B-Antibiotic dosage | 3162.21 | 1 | 3162.21 | 76.35 | < 0.0001 |  |
| C- Shaking time | 1019.82 | 1 | 1019.82 | 24.62 | 0.0002 |  |
| D-pH | 870.17 | 1 | 870.17 | 21.01 | 0.0004 |  |
| AB | 100.72 | 1 | 100.72 | 2.43 | 0.1397 |  |
| AC | 43.20 | 1 | 43.20 | 1.04 | 0.3233 |  |
| AD | 115.16 | 1 | 115.16 | 2.78 | 0.1162 |  |
| BC | 20.70 | 1 | 20.70 | 0.4997 | 0.4905 |  |
| BD | 15.24 | 1 | 15.24 | 0.3680 | 0.5532 |  |
| CD | 195.19 | 1 | 195.19 | 4.71 | 0.0464 |  |
| A² | 5.26 | 1 | 5.26 | 0.1270 | 0.7265 |  |
| B² | 499.71 | 1 | 499.71 | 12.06 | 0.0034 |  |
| C² | 79.77 | 1 | 79.77 | 1.93 | 0.1855 |  |
| D² | 825.88 | 1 | 825.88 | 19.94 | 0.0005 |  |
| **Residual** | 621.28 | 15 | 41.42 |  |  |  |
| Lack of Fit | 621.28 | 10 | 62.13 |  |  |  |
| Pure Error | 0.0000 | 5 | 0.0000 |  |  |  |
| **Cor Total** | 7423.33 | 29 |  |  |  |  |
| **Quadratic model summary statistics**  **R² 0.9163** | **Adjusted R²**  0.8382 | **Predicted R²**  0.5179 | **Adeq**  **Precision**  11.3545 | **Std. Dev.**  6.44 | **Mean**  73.54 | **C.V. %**  8.75 |

| 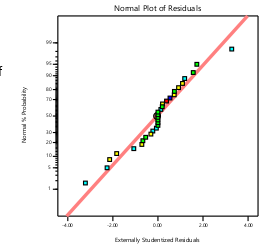 | 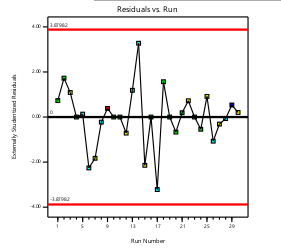 |
| --- | --- |
| **(a)** | **(c)** |
| 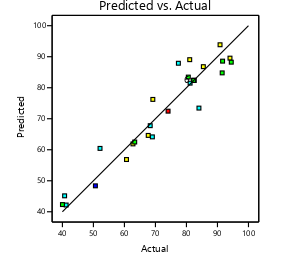 | 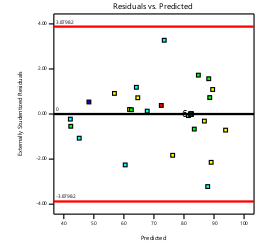 |
| **(b)** | **(d)** |

**Figure S4**. (a) Normal probability plot of the residual, (b) predicted versus actual amounts diagram, (c) residual versus run number, and (d) residual versus predicated amount for the photodegradation performance of CIPF.

| 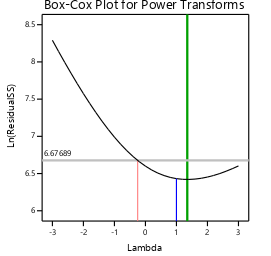 | 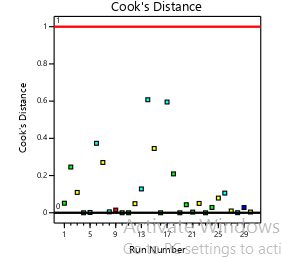 |
| --- | --- |
| (a) | (b) |

### Figure S5. (a) Box–Cox versus Lambda plot for the Power transformation and (b) Cook, S Distance versus Run Number for the photodegradation performance of CIPF.

###
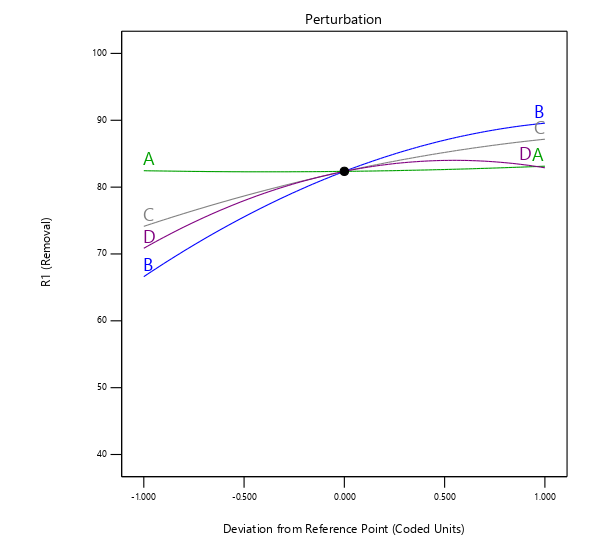


**Figure S6**. The perturbation graph for experiments’ parameters on CIPF removal efficiency under RSM method.

| 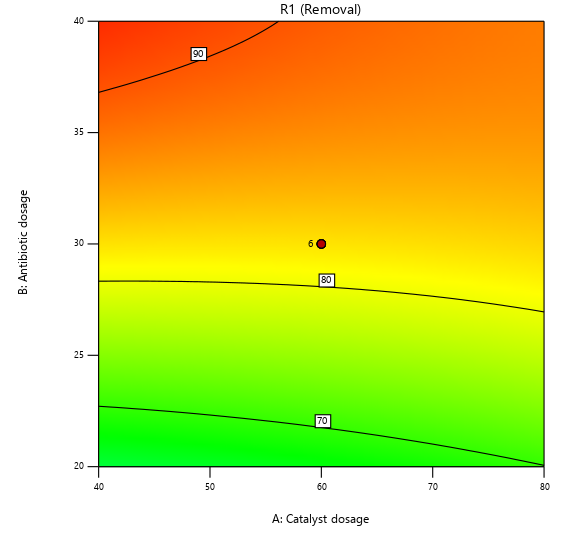 | 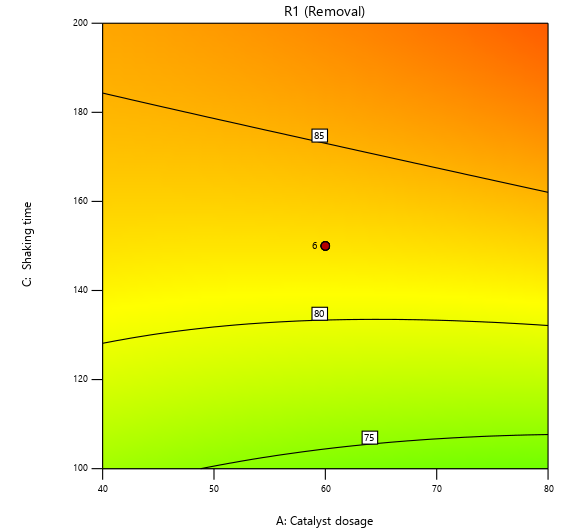 |
| --- | --- |
| 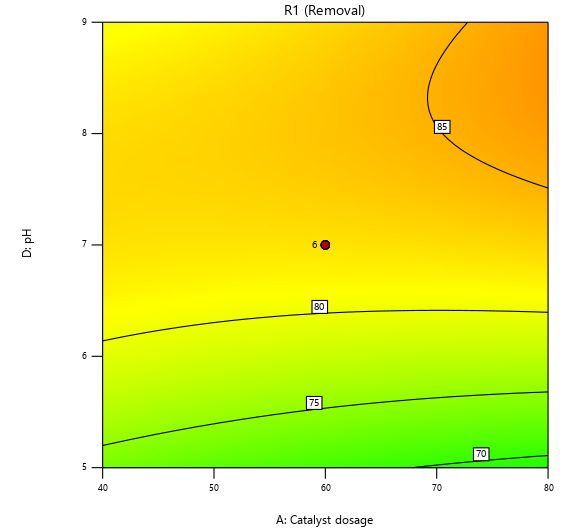 | 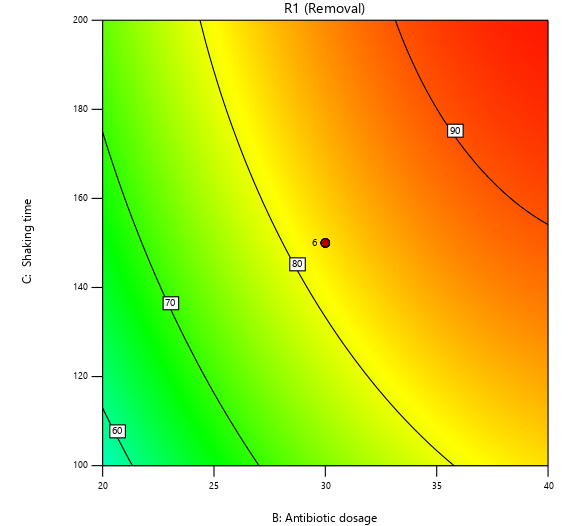 |
| 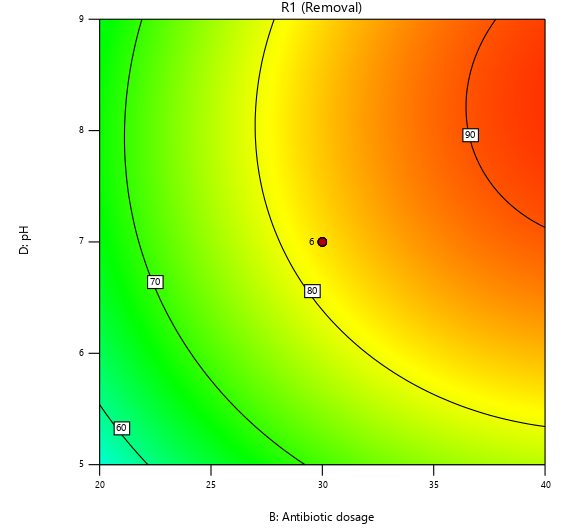 | 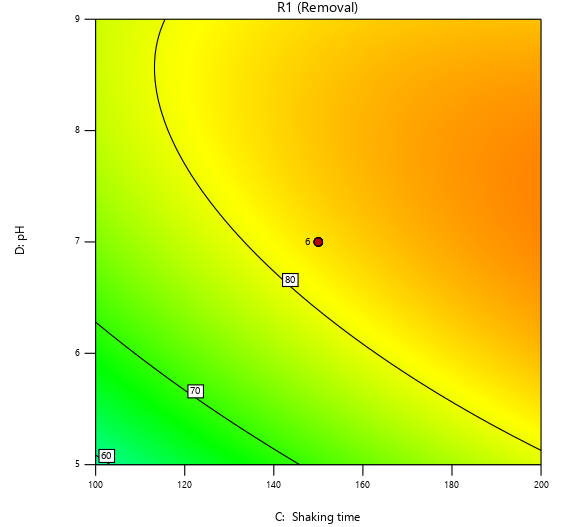 |

**Figure 7S**. 2D contours plots for the photodegradation efficiency of CIPF.

### References

[1] Kalpana, S., Krishnan, S.S., Senthil, T.S. and Elangovan, S.V., 2017. Cobalt doped Zinc oxide nanoparticles for photocatalytic applications. *Journal of Ovonic Research Vol*, *13*(5), pp.263-269.

[2] Poornaprakash, B., Chalapathi, U., Subramanyam, K., Vattikuti, S.P. and Park, S.H., 2020. Wurtzite phase Co-doped ZnO nanorods: Morphological, structural, optical, magnetic, and enhanced photocatalytic characteristics. *Ceramics International*, *46*(3), pp.2931-2939.

[3] Nair, M.G., Nirmala, M., Rekha, K. and Anukaliani, A., 2011. Structural, optical, photo catalytic and antibacterial activity of ZnO and Co doped ZnO nanoparticles. *Materials letters*, *65*(12), pp.1797-1800.

[4] Sutanto, H., Wibowo, S., Arifin, M. and Hidayanto, E., 2017. Photocatalytic activity of cobalt-doped zinc oxide thin film prepared using the spray coating technique. *Materials Research Express*, *4*(7), p.076409.

[5] Yildirim, O.A., Arslan, H. and Sönmezoğlu, S., 2016. Facile synthesis of cobalt-doped zinc oxide thin films for highly efficient visible light photocatalysts. *Applied Surface Science*, *390*, pp.111-121.

[6] Kuriakose, S., Satpati, B. and Mohapatra, S., 2014. Enhanced photocatalytic activity of Co doped ZnO nanodisks and nanorods prepared by a facile wet chemical method. *Physical Chemistry Chemical Physics*, *16*(25), pp.12741-12749.

[7] Mohammed, L.H., Gulbagca, F., Tiri, R.N.E., Aygun, A., Bekmezci, M. and Sen, F., 2023. Hydrothermal-assisted synthesis of Co-doped ZnO nanoparticles catalyst for sodium borohydride dehydrogenation and photodegradation of organic pollutants in water. *Chemical Engineering Journal Advances*, *14*, p.100495.
